# Time to harmonize national ambient air quality standards

**DOI:** 10.1007/s00038-017-0952-y

**Published:** 2017-02-27

**Authors:** Meltem Kutlar Joss, Marloes Eeftens, Emily Gintowt, Ron Kappeler, Nino Künzli

**Affiliations:** 1 Swiss Tropical and Public Health Institute, P.O. Box 4002, Basel, Switzerland; 2 University of Basel, Basel, Switzerland

**Keywords:** Air quality, Air pollution, Policy, Limit values, Standards, Particulate matter

## Abstract

**Objectives:**

The World Health Organization has developed ambient air quality guidelines at levels considered to be safe or of acceptable risk for human health. These guidelines are meant to support governments in defining national standards. It is unclear how they are followed.

**Methods:**

We compiled an inventory of ambient air quality standards for 194 countries worldwide for six air pollutants: PM_2.5_, PM_10_, ozone, nitrogen dioxide, sulphur dioxide and carbon monoxide. We conducted literature and internet searches and asked country representatives about national ambient air quality standards.

**Results:**

We found information on 170 countries including 57 countries that did not set any air quality standards. Levels varied greatly by country and by pollutant. Ambient air quality standards for PM_2.5_, PM_10_ and SO_2_ poorly complied with WHO guideline values. The agreement was higher for CO, SO_2_ (10-min averaging time) and NO_2_.

**Conclusions:**

Regulatory differences mirror the differences in air quality and the related burden of disease around the globe. Governments worldwide should adopt science based air quality standards and clean air management plans to continuously improve air quality locally, nationally, and globally.

**Electronic supplementary material:**

The online version of this article (doi:10.1007/s00038-017-0952-y) contains supplementary material, which is available to authorized users.

## Introduction

Ambient air pollution is hazardous to human health and caused an estimated 3.7 million premature deaths worldwide in 2012 (WHO 2014). A minor proportion of this burden is a consequence of acute effects related to exposure experienced during a few days, leading to hospitalization due to cardiorespiratory health problems and premature death (Brook et al. 2010). The major proportion of the burden is due to the long-term effects of poor air quality leading to chronic pathologies, such as atherosclerosis (Künzli et al. 2010), asthma in children (Hwang et al. 2015), or lung cancer in adults (Hamra et al. 2015) and decreased life expectancy due to cardiorespiratory diseases (Heroux et al. 2015). Others observed lower birth weight due to exposure to air pollution of pregnant mothers (Stieb et al. 2012). More recently, diabetes (Eze et al. 2015), cognitive development of children (Sunyer et al. 2015) and dementia (Peters et al. 2015) are discussed as additional adverse effects.

The WHO Regional Office for Europe gathered scientific evidence on health effects of various air pollutants for the first time in 1987. The aim of the WHO guideline was to advise governments on setting legally binding air quality standards at levels considered to be safe or of acceptable risk (WHO Regional Office for Europe 1987). In 2005, these guidelines were revised and updated for the “classical” outdoor air pollutants PM_2.5_ (suspended particles smaller than 2.5 µm in size), PM_10_ (suspended particles smaller than 10 µm), ozone (O_3_), nitrogen dioxide (NO_2_) and sulphur dioxide (SO_2_) (WHO Regional Office for Europe 2006). In 2015, the WHO started a process to update the air quality guidelines for these pollutants and for carbon monoxide (CO). In 2005, interim targets were proposed to promote steady progress towards meeting the guideline values. However, epidemiological evidence was unable to support any “thresholds of no effect”. Thus, adverse health effects may occur even at levels below the proposed guideline values (WHO Regional Office for Europe 2006). The most recent completed review of the scientific literature initiated by the WHO indicated not only full confirmation of previous evidence but indication of additional adverse health effects explained by air pollution and likely an underestimation of the related burden (Heroux et al. 2015; WHO Regional Office for Europe 2013). Meanwhile, several studies also confirm the public health benefits of clean air policies that result in improved air quality, as seen in many countries over the past 20 years (Brauer et al. 2016; Gauderman et al. 2015; Imboden et al. 2009; Schikowski et al. 2013; Environmental Protection Agency-Office of Air and Radiation 2011). However, despite knowledge about the benefits of clean air policies following the WHO guideline values, it is unclear if or to what extent those guidelines are followed by national or regional policy makers.

In 2012, Vahlsing and Smith (2012) compiled short-term national air quality standards for PM and SO_2_. They stated that only a few systematic investigations appear to have been conducted to review ambient air quality standards globally. To our knowledge, this is still true today, even though several overviews have been conducted on a regional level (Clean Air Initiative for Asian Cities 2010; Clean Air Institute 2013; Schwela 2012). Our recent compilation of PM air quality standards revealed substantial heterogeneity around the globe (Kuenzli et al. 2015).

In anticipation of the future WHO guideline revision, we aimed at compiling national short- and long-term ambient air quality standards of the classical ambient air pollutants and CO in relation to the WHO guidelines. We further discuss the way forward to harmonize standards with the goal of protecting public health.

## Methods

Between March 2015 and June 2016, we conducted literature searches for official documents on national air pollution legislation for the 194 WHO member states (WHO 2016b). We visited websites of ministries of health, environment or energy, and environmental performance reports by international agencies like World Bank or UNECE. We asked country representatives at conferences and international collaborators, staff and international students from the Swiss Tropical and Public Health Institute for information about national air quality standards and responsible authorities from their native countries. We also assessed several regional overviews (Clean Air Initiative for Asian Cities 2010; Clean Air Institute 2013; Schwela 2012) and the air quality policy catalogue from UNEP (United Nations Environment Programme 2015). If we were not able to locate any direct reference, we cited values from the Airlex database (Universidade de Aveiro: Instituto do Ambiente e Desenvolvimento 2013). We also checked whether EU member states had different values than those proposed by the EU (European Parliament 2008) and registered regions and cities with differing air quality standards from those set by their federal authorities.

For each country, we tabulated ambient air quality standards for different averaging times: PM_2.5_ (24-h and annual average), PM_10_ (24-h and annual average), O_3_ (1-h mean and 8-h average), NO_2_ (1- and 24-h average), SO_2_ (10-min, 24-h and annual average), and CO (15-min, 1-, 8- and 24-h average). References and additional information (where necessary) are included in the table in the supplementary material (Online Resources 1, 2) and on https://www.swisstph.ch/en/projects/ludok/grenzwerte/.

We assumed that no standard was set for pollutants and/or averaging times that went unmentioned in the governmental documents. When more than one standard was defined for a single pollutant, we chose to list the standard that was most relevant for human exposure, e.g. standards for residential areas. The category “other” allowed us to indicate that a standard was set for a similar averaging time or pollutant, but a direct quantitative comparison was not possible. In this regard, PM_2.5_, PM_10_ and total suspended particles (TSP) were considered similar pollutants, as were NO_2_ and NO_X_. We considered that all averaging times between 1 and 24-h were similarly representative of regulations targeting short-term exposures to NO_2_, O_3_ and SO_2_, and that averaging times ≤1-h were similarly representative of ultra-short-term regulations for SO_2_. For all pollutants, standards set for averaging times >24-h (e.g. 1 month, wintertime) were considered representative for the regulation of long-term conditions. Wherever maximum allowable concentration (MAC) values or maximum permissible concentration (MPC) values were defined, we also listed these as “other” under the respective averaging time(s).

We quantified the number of countries that have set ambient air quality standards for at least one pollutant and averaging time for each region of the world, following the WHO’s geographical region classification (WHO 2016b). We further presented for each pollutant and averaging time how many countries had air quality standards in place, had similar (“other”) standards in place, confirmedly had no regulations in place, or had no information available. We visualized the distribution of air quality standards compared to the WHO guideline values using violin plots and world maps. For these maps we additionally evaluated whether air quality standards applied to geographical areas under the sovereignty of other countries. If no information was available, we assumed that the same standards applied to these areas as for the respective mother-country.

### Results

The complete inventory table of national air quality standards for all pollutants and averaging times is shown in (Online Resource 1). We were able to identify whether air quality standards were set or not for 170 out of 194 countries (Table 1). No standards were defined by 53 (27%) countries for any ambient air pollutant under review. This was especially the case in countries from the Western Pacific Region which includes many small island states (48%), the African region (45%) and the Region of Americas (37%). For Europe, Region of Americas, and Western Pacific the percentage of countries without any information was low (2, 6, and 7%, respectively). We were not able to find any information on regulation for 43% of the Eastern Mediterranean and 19% of the African countries (Table 1).

**Table 1:** Number of countries with and without identifiable information on clean air policies (standard setting) and number of countries that have not set any ambient air quality standard in the six world-regions as defined by the WHO

	Number of countries	Number of countries with standards for at least one pollutant and averaging time	Number of countries without standards	Number of countries without any information
European Region	53	50 (94%)	2 (4%)	1 (2%)
Region of Americas	35	20 (57%)	13 (37%)	2 (6%)
African Region	47	17 (36%)	21 (45%)	9 (19%)
Eastern Mediterranean Region	21	11 (52%)	1 (5%)	9 (43%)
South-East Asia	11	7 (64%)	3 (27%)	1 (9%)
Western Pacific	27	12 (44%)	13 (48%)	2 (7%)
Total	194	117 (60%)	53 (27%)	24 (12%)

**Fig. 1:**
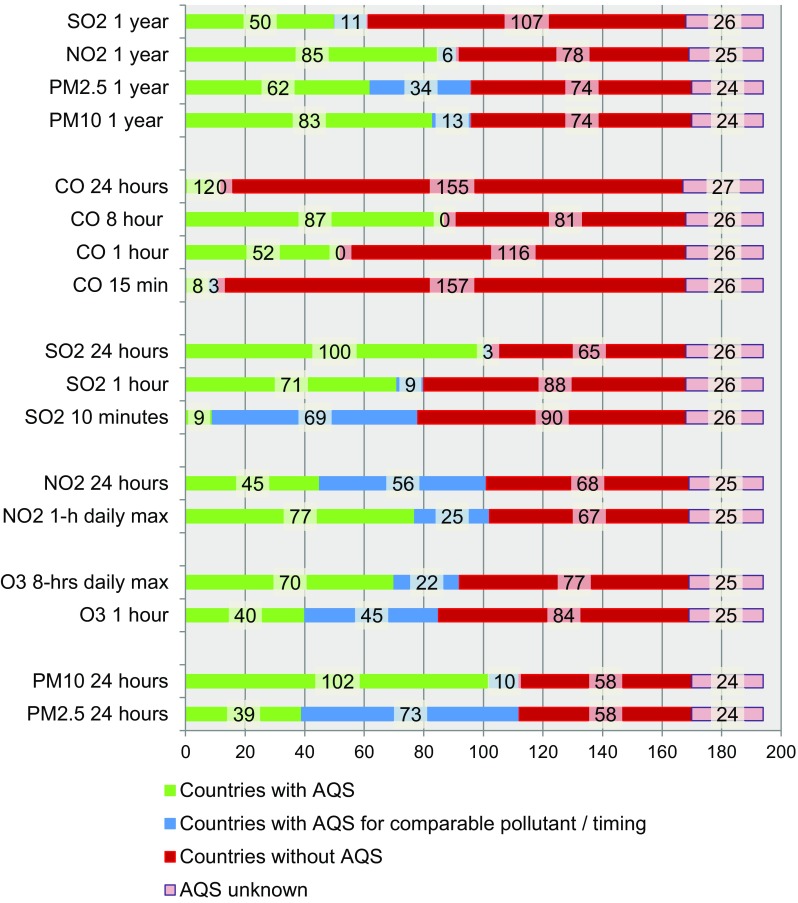
Number of countries with air quality standard (AQS), with comparable standard, without standard and with unknown status regarding regulation of ambient air pollutants for long-term and shorter averaging times (up to 24-h)

Figure 1 provides an overview of the number of countries that have set a standard for a specific pollutant and averaging time, have set another comparable standard, have not set a standard for the specific pollutant or without any information available.

**Fig. 2:**
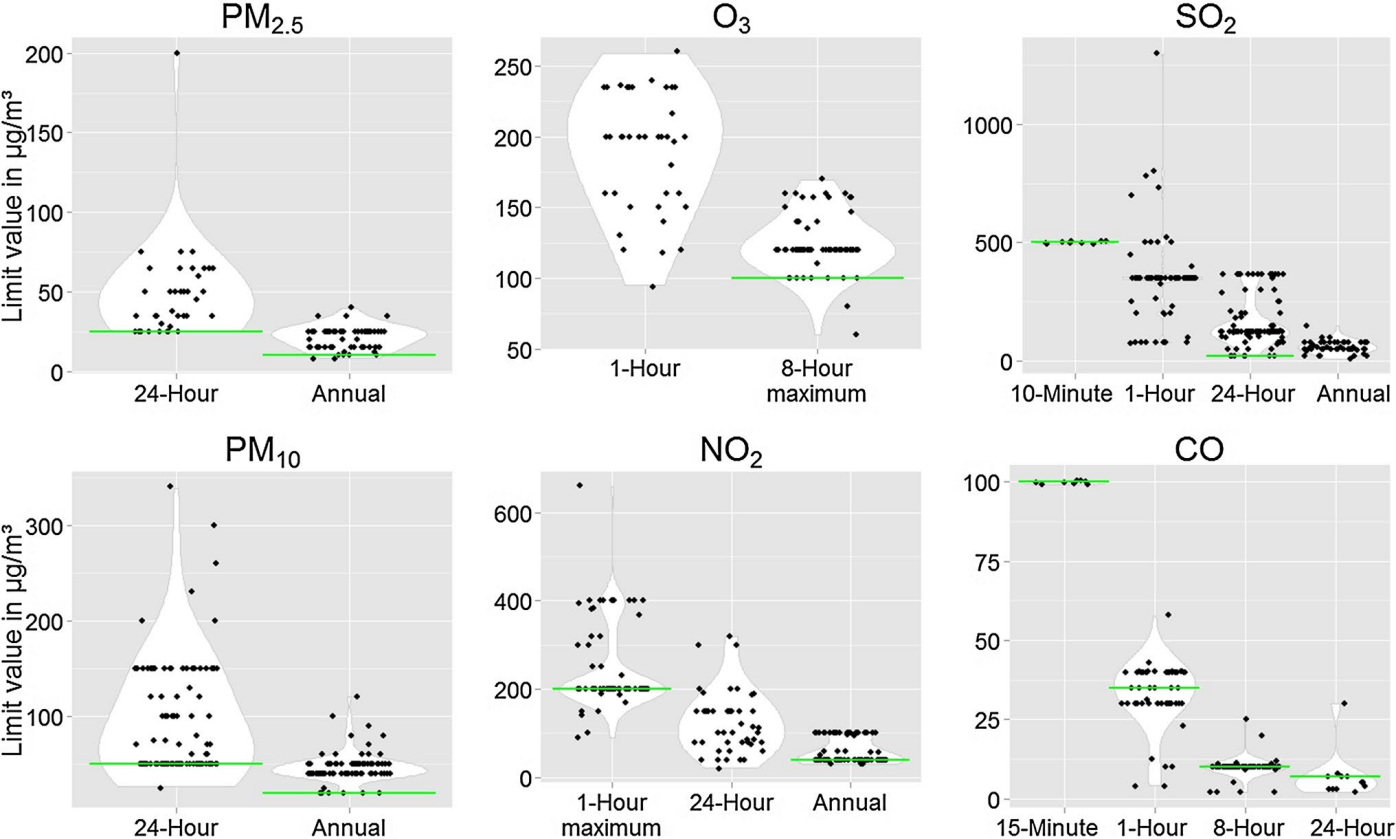
Violin plots showing a fitted distribution and range of national air quality standards for PM_2.5_, PM_10_, O_3_, NO_2_, SO_2_ and CO and for different averaging times. The *grey line* indicates the level of the WHO guideline value. To better show the number of points and distribution, minimal jitter was introduced around the 500 µg/m³ level for the 10-min SO_2_, the 300 µg/m³ level for the 1-h SO_2_ and the 100 µg/m³ level for the 15-min CO plots

**Fig. 3:**
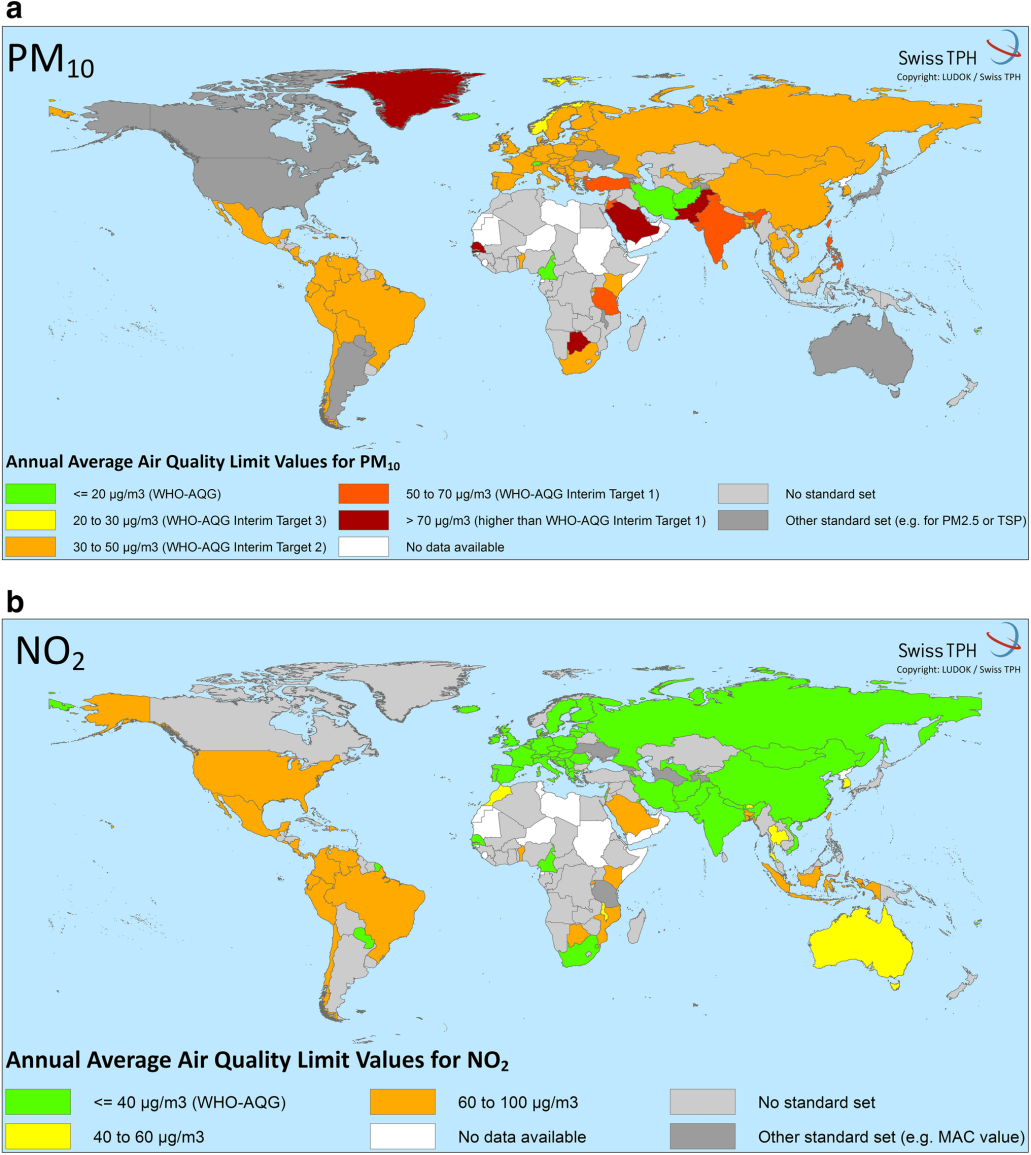
**a, b** World maps of national ambient air quality standards in relation to WHO air quality guideline values (WHO-AQG) and interim targets for annual limit values for PM_10_ and NO_2_

The pollutants most often regulated with short-term standards (averaging time ≤24-h) are ambient daily averages of PM_10_, SO_2,_ and 1-h maximum values for NO_2_. Standards for annual mean concentrations were generally set by fewer countries. Most standards are set at higher levels than those proposed by the WHO as shown in Fig. 2. These violin plots illustrate the distribution and range of national limit values compared to the WHO guideline values. Table 2 shows the number of countries compliant with the WHO guideline indicating that adoption of the WHO guideline values for short-term averaging times is generally higher than for long-term standards. Among countries with standards for 24-h averaging times for PM_2.5_ and PM_10_, 21 and 46%, respectively, met the guideline values. Only 7 and 2% adopted the WHO’s annual mean guideline values for PM_10_ and PM_2.5_, respectively. The recommended 24-h standard for SO_2_ was met by 7% of countries and 16% met the 1-h standards. The agreement was higher for CO, SO_2_ (10-min averaging time) and NO_2_ (58–100%). Figure 3a, b show world maps on the level of compliance with the WHO guideline values and interim targets in different countries for annual mean concentrations of PM_10_ and NO_2_. Maps for level of compliance for annual mean concentrations of PM_2.5_ and SO_2_ can be found in the Online Resource 3 and 4. Maps for short-term averaging times can be found in Online Resource 5 (PM_10_), 6 (PM_2.5_), 7 (SO_2_), 8 (NO_2_), 9 (O_3_), 10 and 11 (CO).

**Table 2:** WHO air quality guideline values (AQG), number of countries with air quality standards (AQS) and percentage of countries compliant with WHO recommendation for the classical pollutants PM_2.5_, PM_10_, ozone, NO_2_, SO_2_ and CO for various short- and long-term averaging times

	Short-term ambient air quality standards													Long-term ambient air quality standards			
	PM_2.5_ 24-h (µg/m^3^)	PM_10_ 24-h (µg/m^3^)	O_3_ 1-h (µg/m^3^)	O_3_ 8-h daily max (µg/m^3^)	NO_2_ 1-h daily (µg/m^3^)mean	NO_2_ 24-h (µg/m^3^)	SO_2_ 10-min (µg/m^3^)	SO_2_ 1-h (µg/m^3^)	SO_2_ 24-h (µg/m^3^)	CO 15-min (mg/m^3^)	CO 1-h (mg/m^3^)	CO 8-h (mg/m^3^)	CO 24-h (mg/m^3^)	PM_10_ 1-year (µg/m^3^)	PM_2.5_ 1-year (µg/m^3^)	NO_2_ 1-year (µg/m^3^)	SO_2_ 1 year (µg/m^3^)
No. of countries with AQS	39	101	40	70	77	45	9	71	100	8	52	87	12	83	62	85	50
Number and (%) of countries compliant	8 (21%)	47 (46%)	n/a	11 (16%)	56 (73%)	n/a	9 (100%)	n/a	7 (7%)	8 (100%)	30 (58%)	81 (93%)	7 (58%)	6 (7%)	6 (2%)	56 (66%)	n/a
WHO AQGs	25	50	None	100	200	None	500	None	20	100	35	10	7	20	10	40	None
WHO Interim target 3	37.5	75	None	None	None	None	None	None		None	None	None	None	30	15	None	None
WHO Interim target 2	50	100	None	None	None	None	None	None	50	None	None	None	None	50	25	None	None
WHO Interim target 1	75	150	None	160	None	None	None	None	125	None	None	None	None	70	35	None	None

Footnote regarding averaging times: 24-h—daily mean, mean of 24-h, 1-h: mean of 1-h values, O_3_ 8-h daily max: daily maximum of running 8-h mean

### Ambient air quality standards for short-term averaging times

The guideline value of 50 µg/m^3^ for 24-h PM_10_ was met by 47 countries including Malawi with an even more stringent standard of 25 µg/m^3^. The six countries Burkina Faso, Botswana, Benin, Senegal, Uzbekistan and Saudi Arabia have set higher standards than the WHO Interim-Target 1 which was set at 150 µg/m^3^. Canada was the only country that did not regulate PM10 but PM2.5, only. Seven countries out of 100 adopted the daily mean WHO guideline value of 20 µg/m^3^ for SO_2_. Regarding NO_2_ standards for 1-h daily maximum 56 out of the 77 countries that have set standards complied at least with the standard proposed by the WHO.

### Ambient air quality standards for long-term periods

NO_2_ was the most often regulated pollutant with 85 countries of which 56 complied with the WHO guideline value set at 40 µg/m^3^ as the annual mean concentration. Lower limits (30 µg/m^3^) were set by Switzerland, Austria and Mongolia. Long-term standards for PM_10_ and/or PM_2.5_ were set by 83 countries. Seven countries regulated PM_2.5_ only without standards for PM_10_ (Australia, Canada, Japan, Malawi, Paraguay, Singapore and the US). The PM_10_ WHO annual guideline value of 20 µg/m^3^ was adopted by six authorities (Afghanistan, Cameroon, Fiji, Iceland, Iran and Switzerland), whereas most countries have set values between 40 and 50 µg/m^3^ up to 120 µg/m^3^. Annual mean PM_2.5_ limits were set by 62 countries at around 25 µg/m^3^ as opposed to the WHO guideline value of 10 µg/m^3^, met by six countries only, namely Australia and Malawi with a 20% lower standard (8 µg/m^3^) and Afghanistan, Cameroon, Canada and Iran.

## Discussion

To our knowledge, this is the first worldwide overview of short-term and long-term ambient air quality standards for O_3_, NO_2_ and CO. We also provide an update on short- and long-term standards set for PM_2.5_, PM_10_ and SO_2_. We were able to compile information about ambient air quality standards from 170 countries from all continents. The standards varied greatly between countries, despite universally generalizable scientific evidence of the substantial and serious health effects of ambient air pollution. The discrepancy in dealing with scientific evidence reflects the diversity in abilities and priorities of policy makers to regulate air quality and to implement policies that aim at reducing air pollution and protecting health. According to the WHO, its guideline values aim at air quality with only little or no effects for human health. As acknowledged by the WHO, our study confirms that it is not only public health concerns and scientific evidence driving the setting of air quality standards but also perceptions about technological feasibility, economic constraints, political pressure and social factors (WHO Regional Office for Europe 2006).

In general, the largest discrepancies in air quality standards are seen between high and low income countries. Vahlsing and Smith observed an inverse correlation between short-term air quality standards and health expenditures of countries. Additionally, countries with high PM_10_ levels have higher standards (Vahlsing and Smith 2012). We confirm this observation comparing PM_10_ standards to actual levels in countries compiled in the WHO Ambient Air Pollution Database (WHO 2016a) (see Online Resources 12–14). This also reflects the WHO’s finding that 98% of cities in low- and middle-income countries with more than 100,000 inhabitants have air quality levels that do not meet the WHO air quality guidelines (WHO 2016a). According to the 2013 global burden of disease (GBD) estimates, ambient air pollution (i.e. particulate matter alone) is one of the top ten causes for the burden of disease in over 50 countries worldwide (G. B. D. Risk Factors Collaborators et al. 2015). The lack of clean air policies and their enforcements pose an additional threat to the health systems and economies of resource constrained countries (G. B. D. Risk Factors Collaborators et al. 2015). Also, the Commission of the European Union still opposes the adoption of the WHO guideline values for PM, despite having invested large amounts of tax money into cutting-edge research, providing evidence of the adverse health effects of air pollution in Europe (Brunekreef et al. 2012, 2015). The resistance to adopt science based standards is unfortunate, given that air pollution is largely preventable and that the costs of the health burden of air pollution are several fold higher than the costs of effective clean air policies (US Environmental Protection Agency-Office of Air and Radiation 2011).

The biggest concerns from a global health perspective are the enormous differences in air quality and the opposite trends seen in the last 20–30 years. Whereas the least polluted countries and regions experienced continued—and very substantial—improvements in air quality due to emission control and other clean air policies, the most polluted regions in the world are challenged by further deteriorations (Brauer et al. 2016). As shown by Wang et al. (2016) using the 1990–2010 trends of ambient PM_2.5_ concentrations, the related attributable mortality decreased some 60% in high income countries, while increasing up to 85% in other regions of the world, e.g. in South Asia.

The current situation is the result of poor air quality governance, uncontrolled economic development and fast urbanization seen in many low- and middle-income countries. In addition, it is a consequence of globally acting companies from high income countries, profiting from exporting heavily polluting industries, products and activities into countries with laxer clean air policies and enforcement than their home countries (Public Eye 2016). This further amplifies the differences in air quality and the related health burden. Moreover, the burden of air pollution related health problems affects the low- and middle-income countries and poverty related inequities in exposure within countries and cities. Those in lower socio-economic positions may often—although not in all cities or regions—reside in more polluted areas such as along busy roads or close to industries than the wealthier people who may afford greener and cleaner neighbourhoods (e.g. Jephcote and Chen 2012).

The hesitation of governments to adopt air quality standards in line with the WHO guideline values and implementing supporting policies poses both a public health and economic threat and jeopardizes the achievement of the Sustainable Development Goals (United Nations 2016; WHO Regional Office for Europe 2016). To reduce this threat and to eliminate the inequity, air quality should become a priority on the political agenda worldwide, as also acknowledged by the World Health Assembly in 2015 in its resolution on air pollution. It urges member states to take into account the WHO ambient and indoor air quality guidelines in the development of multisectoral national responses to air pollution and to carry out measures supporting the aims of those guidelines (WHO 2015). Moreover, air pollution crosses borders, thus, countries with poor clean air policies may also hamper the ability of neighbouring countries to achieve public health oriented standards (Brunekreef et al. 2012).

The increasing inequity provides a strong case to call for the ultimate objective to achieve the same science based standards by all governments of all countries. Air quality standards do not improve air quality per se, but they do set clear and legally binding targets for clean air policies, thus, provide an important framework and guidance for authorities in charge of clean air management. The latter need to tailor policies and plans to local sources, needs, and priorities, thus, the time needed to comply with science based standards as those proposed by the WHO will also vary. All countries should get involved in the constant process of improving air quality and health by setting ambient standards, formulating policies and timelines for milestones, implementing measures, reevaluating achievements in air quality and hurdles jeopardizing further progress. This process—also described as the four stages of the Public Health Action Cycle (assessment, policy formulation, assurance, evaluation) (Rosenbrock 1995)—has been shown to be highly effective in the field of air pollution regulation [see also Kuenzli and Perez (2009)]. The very strong improvements of air quality in former hot spots of smog, such as Los Angeles or London, are vivid examples of the feasibility to drastically improve air quality.

### Limitations and strengths

Even though we have put great effort and diligence in compiling as many national ambient air quality standards as possible, the question remains whether those standards are still valid. For some countries, we were able to directly access the regulatory texts or had personal assistance by local experts. However, for other countries we had to rely on third party information which might be outdated. Moreover, we cannot systematically cover ongoing discussions about possible changes of national regulations, though we are aware of some national dynamics. For example, as observed during the study, countries such as China (TransportPolicy.net 2014) or Switzerland [Eidgenössische Kommission für Lufthygiene (EKL) 2013] have recently set or are in the process of setting stricter air quality targets.

One should also be aware of the limited comparability between apparently equal standards. Although countries might have set equal values into standards, the allowed annual number of exceedances may vary substantially between countries. Or the way concentrations are measured might differ (Brunekreef and Maynard 2008). Thus, “being in compliance” with national legislation may still reflect differences in air quality, and thus health impact (Brunekreef and Maynard 2008). For some countries it is not known whether standards are legally binding or only a guideline. In general, it remains unclear for most countries whether and how compliance with standards is monitored and enforced. More information on national clean air policies can be found in the UNEP Transport Air Quality Policy Catalogue based on research conducted in 2015 (United Nations Environment Programme 2015).

Despite these limitations, our compilation of ambient air quality standards gives a unique overview over the substantial gaps between recommendations for public health oriented science based targets and the adoption of ambient air quality standards by the governments. Countries with long-term experience in improving air quality may share the interdisciplinary expertise in research, technology, management and governance needed to reach more equitable air quality conditions on the global scale.

### Conclusions

Despite strong evidence of the serious health effects of ambient air pollution, we have shown that air quality standards vary greatly among regions and countries. For some pollutants, only few countries are in line with the recommended WHO guideline values proposed to protect people’s health. This is particularly the case for standards regarding particulate matter and SO_2_. These regulatory discrepancies amplify the differences in air quality and related health effects around the globe. To improve air quality locally, nationally, and globally, governments worldwide must identify and overcome the hurdles that currently prevent them from aiming at the targets set by the WHO. They should get involved in a continuous process of developing clean air policies, and monitoring their success, so that ultimately all may achieve the same science based air quality standards and meet the sustainable development goals.

## Electronic supplementary material

Below is the link to the electronic supplementary material.

Supplementary material 1 (PDF 5776 KB)
