# Supplementary material for: Time to harmonize national ambient air quality standards
Source: Int J Public Health. 2017 Feb 27;62(4):453–62. doi: 10.1007/s00038-017-0952-y (PMC5397445; doi:10.1007/s00038-017-0952-y)

Time to harmonize national ambient air quality standards for global health equity

Meltem Kutlar Joss 1,2, Marloes Eeftens 1,2, Emily Gintowt 1,2, Ron Kappeler 1,2, Nino Künzli 1,2

Affiliations :
1 Swiss Tropical and Public Health Institute, Basel, Switzerland
2 University of Basel, Basel, Switzerland
Corresponding author:
Meltem Kutlar Joss, email: meltem.kutlar@unibas.ch, Tel. +41 61 284 88 20

Table of Contents

Online Resource 1. Existing Ambient Air Quality Standards (AQS) by WHO Regions in countries recognized by the United Nations and current WHO Air Quality Guidelines for classical air pollutants (PM2.5, PM10, O3, NO2, SO2 and CO)

Online Resource 2. References to Online Resource 1.

Online Resource 3. World map of national air quality standards for annual mean concentration of PM2.5 in relation to WHO air quality guideline values (WHO-AQG) and interim targets.

Online Resource 4. World map of national air quality standards for annual mean concentration of SO2 in relation to WHO air quality guideline values (WHO-AQG) and interim targets.

Online Resource 5. World map of national air quality standards for daily mean concentration of PM10 in relation to WHO air quality guideline values (WHO-AQG) and interim targets.

Online Resource 7. World map of national air quality standards for daily mean concentration of SO2 in relation to WHO air quality guideline values (WHO-AQG) and interim targets.

Online Resource 7. World map of national air quality standards for daily mean concentration of SO2 in relation to WHO air quality guideline values (WHO-AQG) and interim targets.

Online Resource 8. World map of national air quality standards for 1-hour average concentration of NO2 in relation to WHO air quality guideline values (WHO-AQG) and interim targets.

Online Resource 9. World map of national air quality standards for 8-hour maximum concentration of Ozone in relation to WHO air quality guideline values (WHO-AQG) and interim targets.

Online Resource 10. World map of national air quality standards for 8-hour average concentration of CO in relation to WHO air quality guideline values (WHO-AQG) and interim targets.

Online Resource 11. World map of national air quality standards for 1-hour average concentration of CO in relation to WHO air quality guideline values (WHO-AQG) and interim targets.

Online Resource 12. Existing long-term ambient air quality standards for Particulate Matter by WHO Regions in countries recognized by the United Nations and actual annual mean measurement data from the WHO Global Urban Ambient Air Pollution Database.

Online Resource 13. Comparison of national annual air quality standard set for PM10 to measured annual mean concentrations for the year 2013.

Online Resource 14. Comparison of national annual air quality standard set for PM10 to highest measurement of annual concentration of PM10 in 2013.

Time to harmonize national ambient air quality standards for global health equity

Meltem Kutlar Joss 1,2, Marloes Eeftens 1,2, Emily Gintowt 1,2, Ron Kappeler 1,2, Nino Künzli 1,2

Affiliations :
1 Swiss Tropical and Public Health Institute, Basel, Switzerland
2 University of Basel, Basel, Switzerland
Corresponding author:

Meltem Kutlar Joss, email: meltem.kutlar@unibas.ch, Tel. +41 61 284 88 20

Online Resource 1. Existing Ambient Air quality Standards (AAQSs) by WHO Regions in countries recognized by the United Nations and current WHO Air Quality Guidelines for classical air pollutants (PM2.5, PM10, O3, NO2, SO2 and CO)

	**Pariculate matter <2.5 um (PM2.5) µg/m3**				**Particulate matter < 10 um (PM10) µg/m3**			
	PM2.5 24 hours	PM2.5 1 year	PM2.5 other	year set, revised, or published	PM10 24 hours	PM10 1 year	PM10 other	year set, revised, or published
**WHO AQGs**	25	10		2006	50	20		2006
**WHO REGIONS AQSs**								
**European Region**								
**EU Directive 2008/50/EC**	N/A	25		2008	50	40		2008
Austria	other	25		2015	50	40		2010
Belgium	other	25		!	50	40		!
Bulgaria	N/A	N/A		2011	N/A	N/A		2011
Croatia	other	25		2010/2014	50	40		2010/2014
Cyprus	other	25			50	40		
Czech Republic	other	25			50	40		
Denmark	other	other		2010	50	40		2010
Estonia	other	25			50	40		
Finland	other	25		2011	50	40		2011
France	other	other		2012	50	40		2012
Germany	other	other		2002	50	40		2002
Greece	other	25			50	40		
Hungary	other	25			50	40		
Ireland	other	other		2011	50	40		2011
Italy	other	25		2010	50	40		2010
Latvia	other	25		2007	50	40		2007
Lithuania	other	25		2015	50	40		2005
Luxemburg	other	25		2011	50	40		2011
Malta	other	25		2015	50	40		2015
The Netherlands	other	25		<2006	50	40		<2006
Poland	other	25		2012	50	40		2012
Portugal	other	25			50	40		
Romania	other	25			50	40		
Slovakia	other	25			50	40		
Slovenia	other	25			50	40		
Spain	other	25		2011	50	40		2011
Sweden	other	20		2015	50	40		2010
United Kingdom	other	25	Scotland 12 (1y)	2010	50	40		2010

	Pariculate matter <2.5 um (PM2.5) µg/m3				Particulate matter < 10 um (PM10) µg/m3			
	PM2.5 24 hours	PM2.5 1 year	PM2.5 other	year set, revised, or published	PM10 24 hours	PM10 1 year	PM10 other	year set, revised, or published
Albania	other	other		2003	150	50		2003
Andorra	other	25		2009	50	40		2009
Armenia	35	other	Dust MPC 0.15mg/m3 =1y?	2006	60	other	Dust MPC 0.15mg/m3 =1y?	2006
Azerbaijan	other	N/A	TSP 150 MAC 24h	2011	other	N/A	TSP 150 MAC 24h	2011
Belarus	25	15		2010	50	40		2010
Bosnia and Herzegovina	other	other	25 (1y) starting2021	2012	50	40		2012
Georgia	N/A	other	TSP MAC 150 (1y)	2001	N/A	other	TSP MAC 150 (1y)	2001
Iceland	other	other		2010	50	20		2010
Israel	37.5	25		2014	130	50		2014
Kazakhstan	35	N/A		2012	60	N/A		2012
Kyrgyzstan	other	N/A	PM 24h 150	!	other	N/A	PM 24h 150	!
Monaco								
Montenegro	25	20		2015	50	40		2015
Norway	other	15		2007	50	25		2007
Republic of Moldova	N/A	N/A			N/A	N/A		
Russian Federation	35	25	MAC 160	!	60	40	MAC 300	!
San Marino	N/A	N/A		2012	N/A	N/A		2012
Serbia	other	25		2009	50	40		2009
Switzerland	other	other	PM2.5 (1y) under development	2015	50	20		2015
Tajikistan	other	other	TSP MAC 150	2010	other	other	TSP MAC 150	2010
rmer Yugoslav Republic of Macedonia	other	other		2011	50	40		2011
Turkey	other	other		2008	100	60		2008
Turkmenistan	other	N/A	TSP 150 (24h) MAC 500	1996	other	N/A	TSP 150 (24h) MAC 500	1996
Ukraine	N/A	other	MAC 150 PM (1y)	2001	N/A	other	MAC 150 PM (1y)	2001
Uzbekistan	other	other		2010	300	50		2010
**Region of the Americas**								
Antigua and Barbuda	N/A	N/A			N/A	N/A		
Argentina	other	other	en suspension 150 ug/m3 (1 Month)	2013	other	other	s en suspension 150 ug/m3 (1 Month)	2013
Bahamas	other	other			50	40		
Barbados	N/A	N/A		2000	N/A	N/A		2000
Belize	N/A	N/A	only emission limits	2003	N/A	N/A		2003
Bolivia (Plurinational State of)	other	other	city region with different AQS	2013	150	50	city region with different values	2013
Brazil	other	other		2013	150	50		2013
Canada	28	10		2015	other	other	city region with different values	2015
Chile	50	20		2013	150	50		2013
Colombia	50	25		2013	150	50		2013
Costa Rica	other	other		2013	150	50		2013
Cuba	N/A	N/A		2013	N/A	N/A		2013
Dominica	N/A	N/A		2013	N/A	N/A		2013
Dominican Republic	65	15		2003	150	50		2003
Ecuador	65	15		2013	150	50		2013
El Salvador	65	15		2013	150	50		2013
Grenada								
Guatemala	N/A	N/A			N/A	N/A		
Guyana	N/A	N/A		2000	N/A	N/A		2000
Haiti	N/A	N/A			N/A	N/A		
Honduras	N/A	N/A			N/A	N/A		

	Pariculate matter <2.5 um (PM2.5) µg/m3				Particulate matter < 10 um (PM10) µg/m3			
	PM2.5 24 hours	PM2.5 1 year	PM2.5 other	year set, revised, or published	PM10 24 hours	PM10 1 year	PM10 other	year set, revised, or published
Jamaica	other	other		2013	150	50		2013
Mexico	45	12		2014	75	40		2014
Nicaragua	other	other		2013	150	50		2013
Panama	other	other		2013	150	50		2013
Paraguay	30	15		2015	150	other		2015
Peru	25	other		2013	150	50		2013
Saint Kitts and Nevis	N/A	N/A			N/A	N/A		
Saint Lucia	N/A	N/A		2005	N/A	N/A		2005
Saint Vincent and the Grenadines								
Suriname	N/A	N/A			N/A	N/A		
Trinidad and Tobago	65	15		2014	75	50		2014
United States of America	35	12		2016	150	other	city region with different AQS	2016
Uruguay	N/A	N/A			N/A	N/A		
Venezuela (Bolivarian Republic of)	other	other		2013	150	50		2013
**African Region**								
Algeria	other	N/A		2006	other	N/A	fine particles in suspension 80ug/m3	2006
Angola	N/A	N/A			N/A	N/A		
Benin	other	other		2001	230	50		2001
Botswana	other	other		2000	200	100	PM=PM10?	2000
Burkina Faso	200	N/A	PM2.5 200-300 24h	2000	200	N/A	PM10 200-300 24h	2000
Burundi	N/A	N/A			N/A	N/A		
Cameroon	25	10			50	20		
Cabo Verde								
Central African Republic	N/A	N/A			N/A	N/A		
Chad	N/A	N/A		2016	N/A	N/A		2016
Comoros								
Congo	N/A	N/A			N/A	N/A		
Côte d'Ivoire	N/A	N/A		2015	N/A	N/A		2015
Democratic Republic of the Congo	N/A	N/A			N/A	N/A		
Equatorial Guinea								
Eritrea	N/A	N/A			N/A	N/A		
Ethiopia	N/A	N/A			N/A	N/A		
Gabon	N/A	N/A			N/A	N/A		
Gambia	other	N/A			50	N/A		
Ghana	other	N/A		2016	70	N/A		2016
Guinea	N/A	N/A			N/A	N/A		
Guinea-Bissau								
Kenya	75	35	s, none defined for residential areas	2014	100	50		2014
Lesotho	N/A	N/A			N/A	N/A		
Liberia	N/A	N/A			N/A	N/A		
Madagascar	N/A	N/A			N/A	N/A		
Malawi	other	8		2005	25	other	500TSP (1y)	2005
Mali	N/A	N/A			N/A	N/A		
Mauritania								
Mauritius	other	other	TSP 150 (24h) 50 (y)	1998	other	other	TSP 150 (24h) 50 (y)	1998
Mozambique	other	N/A		2005	other	N/A	TSP(24h) 200	2005
Namibia	N/A	N/A			N/A	N/A		

	Pariculate matter <2.5 um (PM2.5) µg/m3				Particulate matter < 10 um (PM10) µg/m3			
	PM2.5 24 hours	PM2.5 1 year	PM2.5 other	year set, revised, or published	PM10 24 hours	PM10 1 year	PM10 other	year set, revised, or published
Niger								
Nigeria	N/A	N/A			N/A	N/A		
Rwanda	other	other		2014	100	50	TSP 140 (1y) 200(24h)	2014
Sao Tome and Principe								
Senegal	other	other		2003	260	80		2003
Seychelles	N/A	N/A			N/A	N/A		
Sierra Leone								
South Africa	65	25		2009	120	50		2009
South Sudan								
Swaziland	other	N/A		2012	50	N/A		2012
Togo	N/A	N/A			N/A	N/A		
Uganda	N/A	N/A			N/A	N/A		
United Republic of Tanzania	other	other		2007	other	60	0.1 ug/Nm3 (daily)	2007
Zambia	other	N/A		1996	70	N/A		1996
Zimbabwe	N/A	N/A			N/A	N/A		
**Eastern Mediterranean Region**								
Afghanistan	25	10		2010	50	20		2010
Bahrain								
Djibouti								
Egypt	other	N/A		1994	70	N/A		1994
Iran (Islamic Republic of)	25	10		2011	50	20		2011
Iraq	N/A	N/A			N/A	N/A		
Jordan	65	15		!	120	70	TSP 75 (1y) 260 (24h)	!
Kuwait	35	15		!	150	90		!
Lebanon	other	N/A		!	80	N/A	TSP 120 (24h)	!
Libya								
Morocco	other	N/A		2009	50	N/A		2009
Oman								
Pakistan	35	15	15 (1h)	2010	150	120		2010
Qatar								
Saudi Arabia	35	15		!	340	80		!
Somalia								
Sudan								
Syrian Arab Republic	other	N/A		2011	70	N/A		2011
Tunisia	N/A	N/A		<2009	N/A	N/A	TSP 550 (24h)	<2009
United Arab Emirates								
Yemen								
**South-East Asia**								
Bangladesh	65	15		2005	150	50		2005
Bhutan	other	other			100	60		
emocratic People's Republic of Korea								
India	60	40		2009	100	60		2009
Indonesia	other	N/A		2010	150	N/A		2010
Maldives	N/A	N/A			N/A	N/A		
Myanmar	N/A	N/A			N/A	N/A		2014
Nepal	other	N/A		2003	120	N/A		2003
Sri Lanka	50	25		2008	100	50		2008

	Pariculate matter <2.5 um (PM2.5) µg/m3				Particulate matter < 10 um (PM10) µg/m3			
	PM2.5 24 hours	PM2.5 1 year	PM2.5 other	year set, revised, or published	PM10 24 hours	PM10 1 year	PM10 other	year set, revised, or published
Thailand	50	25		2010	120	50		2010
Timor-Leste	N/A	N/A			N/A	N/A		
**Western pacific**								
Australia	25	8		2008	50	other		2008
Brunei Darussalam	N/A	N/A			N/A	N/A		
Cambodia	N/A	N/A		2000	N/A	N/A		2000
China	75	35	city region with different values	2014	150	50	city region with different values	2014
Cook Islands								
Fiji	other	other			50	20		
Japan	35	15		!	100	other		!
Kiribati	N/A	N/A			N/A	N/A		
Lao People's Democratic Republic	N/A	N/A			N/A	N/A		
Malaysia	75	35		2015	150	50		2015
Marshall Islands	N/A	N/A			N/A	N/A		
Micronesia (Federated States of)	N/A	N/A			N/A	N/A		
Mongolia	50	25		2007	100	50		2007
Nauru	N/A	N/A			N/A	N/A		
New Zealand	other	N/A		2011	50	N/A		2011
Niue	N/A	N/A			N/A	N/A		
Palau	N/A	N/A			N/A	N/A		
Papua New Guinea	N/A	N/A		2010	N/A	N/A		2010
Philippines	other	other		2014	150	60	TSP 90(Y) TSP 230 (24h)	2014
Republic of Korea	50	25		2013	100	50		2013
Samoa	N/A	N/A			N/A	N/A		
Singapore	25	15	lower targets by 2020	2014	150	other	lower targets by 2020	2014
Solomon Islands	N/A	N/A			N/A	N/A		
Tonga	N/A	N/A		2015	N/A	N/A		2015
Tuvalu	N/A	N/A			N/A	N/A		
Vanuatu (Palestine)								
Viet Nam	50	25		2013	150	50		2013
**OTHER**								
Gibraltar	N/A	25		2010	50	40		2010
St Helena	N/A	N/A		2012	N/A	N/A		2012
Liechtenstein	other	other		2008	50	20		2008
Taiwan	35	15		2016	125	65		2012

	Ozone (O3) µg/m3				Nitrogen dioxide (NO2) µg/m3				
	O3 1 hour	O3 8-hr daily max	other	year set, revised, or published	NO2 1-h	NO2 24 hours	NO2 1 year	NO2 other	year set, revised, or published
**WHO AQGs**		100		2006	200		40		2006
**WHO REGIONS AQSs**									
**European Region**									
**EU Directive 2008/50/EC**	N/A	120		2008	200	other	40		2008
Austria	other	other	Target value 8hmax 120	2010	200	other	30	24h target 80	2012
Belgium	other	120		!	200	other	40		!
Bulgaria	N/A	N/A		2011	N/A	N/A	N/A		2011
Croatia	other	120	180 (1h) info threshold	2010/2014	200	other	40	Nox 30	2010/2014
Cyprus	other	120			200	other	40		
Czech Republic	other	120			200	other	40		
Denmark	other	120		2010	200	other	40		2010
Estonia	other	120			200	other	40		
Finland	other	120		2011	200	other	40		2011
France	other	120		2012	200	other	40	Nox 30(Y)	2012
Germany	N/A	N/A		2002	200	other	40	Nox 30 (y)	2002
Greece	other	120			200	other	40		
Hungary	other	120			200	other	40		
Ireland	other	other	0 (1h) information threshold	2011	200	other	40	NOx 30 (1y)	2011
Italy	N/A	N/A		2010	200	other	40	NOx 30 (1y)	2010
Latvia	other	120			200	other	40		
Lithuania	other	120		2010	200	other	40	NOx 30 (1y)	2010
Luxemburg	other	120		2011	200	other	40		2011
Malta	other	120		2015	200	other	40		2015
The Netherlands	other	120		<2006	200	other	40		<2006
Poland	other	other	alert value 120 (8h)	2012	200	other	40		2012
Portugal	other	120			200	other	40		
Romania	other	120			200	other	40		
Slovakia	other	120			200	other	40		
Slovenia	other	120			200	other	40	Nox 30	
Spain	other	120		2011	200	other	40		2011
Sweden	other	120	180 information threshold	2010	90	60	40		2010
United Kingdom	other	100		2010	200	other	40	Nox 30 (y)	2010

	Ozone (O3) µg/m3				Nitrogen dioxide (NO2) µg/m3				
	O3 1 hour	O3 8-hr daily max	other	year set, revised, or published	NO2 1-h	NO2 24 hours	NO2 1 year	NO2 other	year set, revised, or published
Albania	N/A	N/A	65 (1y)	2003	250	other	40	4hmax 95	2003
Andorra	other	120		2009	200	other	40	NOx 30 (annual)	2009
Armenia	N/A	N/A		2006	other	other	other	1 time 85 (24h?)MPC 40 (1y?)	2006
Azerbaijan	N/A	N/A		2011	other	40	N/A	NOx MAC40	2011
Belarus	other	120		2010	N/A	N/A	N/A		2010
Bosnia and Herzegovina	other	120		2012	200	85	40	Nox 30(1y)	2012
Georgia	N/A	N/A		2001	N/A	N/A	other	MAC 40 (1y)	2001
Iceland	other	120		2003	N/A	N/A	40	NOx 30 (annual)	2002
Israel	other	140		2014	other	200	40		2014
Kazakhstan	N/A	N/A		2012	other	40	N/A		2012
Kyrgyzstan	N/A	other	30 (24h) MAC 160	!	other	40	N/A	MAC 85	!
Monaco									
Montenegro	other	N/A	180 (1h) info threshold	2015	200	other	40		2015
Norway	other	120		2007	200	other	N/A		2007
Republic of Moldova	N/A	N/A			N/A	N/A	N/A		
Russian Federation	other	other	30 24h	!	other	N/A	40	85 (20min)	!
San Marino	N/A	N/A		2012	N/A	N/A	N/A		2012
Serbia	N/A	N/A		2009	150	85	40		2009
Switzerland	120	other		2015	other	80	30		2015
Tajikistan	N/A	N/A		2010	N/A	other	other	MAC 40	2010
rmer Yugoslav Republic of Macedonia	other	120		2011	200	other	40		2011
Turkey	N/A	N/A		2008	300	other	N/A		2008
Turkmenistan	N/A	N/A		1996	other	40	other	MAC 85	1996
Ukraine	N/A	N/A		2001	N/A	other	other	MAC40	2001
Uzbekistan	N/A	N/A		2010	other	60	40		2010
**Region of the Americas**									
Antigua and Barbuda	N/A	N/A			N/A	N/A	N/A		
Argentina	196	other	city region with diff. values	2013	other	other	N/A	0.45 ppm NOx (1h)	2013
Bahamas	200	110			200	N/A	40		
Barbados	N/A	N/A		2000	N/A	N/A	N/A		2000
Belize	N/A	N/A		2003	N/A	N/A	N/A		2003
Bolivia (Plurinational State of)	236	other	city region with diff. values	2013	400	150	N/A	city region with diff. AQS	2013
Brazil	160	other		2013	320	other	100		2013
Canada	N/A	135	city region with diff. values	2015	N/A	N/A	N/A	city region with diff. AQS	2013

	Ozone (O3) µg/m3				Nitrogen dioxide (NO2) µg/m3				
	O3 1 hour	O3 8-hr daily max	other	year set, revised, or published	NO2 1-h	NO2 24 hours	NO2 1 year	NO2 other	year set, revised, or published
Chile	N/A	120		2013	400	other	100		2013
Colombia	120	80		2013	200	150	100		2013
Costa Rica	160	other		2013	400	other	100		2013
Cuba	N/A	N/A		2013	N/A	N/A	N/A		2013
Dominica	N/A	N/A		2013	N/A	N/A	N/A		2013
Dominican Republic	235	160		2003	400	300	100		2003
Ecuador	160	120		2013	other	150	100		2013
El Salvador	235	120		2013	other	150	100		2013
Grenada									
Guatemala	N/A	N/A			N/A	N/A	N/A		
Guyana	N/A	N/A		2000	N/A	N/A	N/A		2000
Haiti	N/A	N/A			N/A	N/A	N/A		
Honduras	N/A	N/A			N/A	N/A	N/A		
Jamaica	235	other		2013	100	other	N/A		2013
Mexico	216	157		2014	395	other	100		2014
Nicaragua	235	160		2013	400	other	100		2013
Panama	235	157		2013	other	150	100		2013
Paraguay	other	120		2015	200	other	40		2015
Peru	other	120		2013	200	other	100		2013
Saint Kitts and Nevis	N/A	N/A			N/A	N/A	N/A		
Saint Lucia	N/A	N/A		2005	N/A	N/A	N/A		2005
Saint Vincent and the Grenadines									
Suriname	N/A	N/A			N/A	N/A	N/A		
Trinidad and Tobago	other	120		2014	200	other	40		2014
United States of America	other	140	city region with diff. AQS	2016	188	other	100	city region with diff. AQS	2016
Uruguay	N/A	N/A			N/A	N/A	N/A		
Venezuela (Bolivarian Republic of)	200	160		2013	367	300	100		2013
**African Region**									
Algeria	N/A	other	200 (?h)	2006	other	other	N/A	200 (?h)	2006
Angola	N/A	N/A			N/A	N/A	N/A		
Benin	N/A	160	=0.08ppm(8h)	2001	other	150	100		2001
Botswana	235	157		2000	400	200	100		2000
Burkina Faso	150	150	0-200 (1h),150-300 8hmax	2000	170	other	N/A		2000
Burundi	N/A	N/A			N/A	N/A	N/A		

	Ozone (O3) µg/m3				Nitrogen dioxide (NO2) µg/m3				
	O3 1 hour	O3 8-hr daily max	other	year set, revised, or published	NO2 1-h	NO2 24 hours	NO2 1 year	NO2 other	year set, revised, or published
Cameroon	N/A	100			200	N/A	40		
Cabo Verde									
Central African Republic	N/A	N/A			N/A	N/A	N/A		
Chad	N/A	N/A		2016	N/A	N/A	N/A		2016
Comoros									
Congo	N/A	N/A			N/A	N/A	N/A		
Côte d'Ivoire	N/A	N/A		2015	N/A	N/A	N/A		2015
Democratic Republic of the Congo	N/A	N/A			N/A	N/A	N/A		
Equatorial Guinea									
Eritrea	N/A	N/A			N/A	N/A	N/A		
Ethiopia	N/A	N/A			N/A	N/A	N/A		
Gabon	N/A	N/A			N/A	N/A	N/A		
Gambia	N/A	N/A			N/A	N/A	40		
Ghana	N/A	N/A		2016	200	60	N/A		2016
Guinea	N/A	N/A			N/A	N/A	N/A		
Guinea-Bissau									
Kenya	200	120		2014	382	191	95		2014
Lesotho	N/A	N/A			N/A	N/A	N/A		
Liberia	N/A	N/A			N/A	N/A	N/A		
Madagascar	N/A	N/A			N/A	N/A	N/A		
Malawi	260	other	80 (4h)	2005	140	other	60		2005
Mali	N/A	N/A			N/A	N/A	N/A		
Mauritania									
Mauritius	94	other		1998	other	187	N/A		1998
Mozambique	160	other	24h 50 1y 70	2005	400	200	100		2005
Namibia	N/A	N/A			N/A	N/A	N/A		
Niger									
Nigeria	N/A	N/A			N/A	N/A	N/A		
Rwanda	240	other	(8h) 1.25 ppm too high?	2014	380	190	96	150 (1month)	2014
Sao Tome and Principe									
Senegal	other	120		2003	200	other	40		2003
Seychelles	N/A	N/A			N/A	N/A	N/A		
Sierra Leone									
South Africa	other	120		2009	200	other	40		2009

	Ozone (O3) µg/m3				Nitrogen dioxide (NO2) µg/m3				
	O3 1 hour	O3 8-hr daily max	other	year set, revised, or published	NO2 1-h	NO2 24 hours	NO2 1 year	NO2 other	year set, revised, or published
South Sudan									
Swaziland	N/A	N/A		2012	N/A	N/A	40		2012
Togo	N/A	N/A			N/A	N/A	N/A		
Uganda	N/A	N/A			N/A	N/A	N/A		
United Republic of Tanzania	other	120		2007	150	120	other	0.1 ug/m3 annual mean	2007
Zambia	N/A	N/A		1996	400	150	N/A		1996
Zimbabwe	N/A	N/A			N/A	N/A	N/A		
**Eastern Mediterranean Region**									
Afghanistan	N/A	100		2010	200	other	40		2010
Bahrain									
Djibouti									
Egypt	200	120		1994	400	150	N/A		1994
Iran (Islamic Republic of)	other	100		2011	N/A	N/A	40		2011
Iraq	N/A	N/A			N/A	N/A	N/A		
Jordan	N/A	N/A		!	N/A	N/A	N/A		!
Kuwait	other	100		!	200	other	40		!
Lebanon	150	100		!	200	150	100		!
Libya									
Morocco	N/A	N/A		2009	other	20	50		2009
Oman									
Pakistan	130	other		2010	other	80	40		2010
Qatar									
Saudi Arabia	235	157		!	660	other	100		!
Somalia									
Sudan									
Syrian Arab Republic	N/A	N/A		2011	200	N/A	N/A		2011
Tunisia									
United Arab Emirates									
Yemen									
**South-East Asia**									
Bangladesh	235	157		2005	N/A	N/A	100		2005
Bhutan	N/A	N/A			N/A	80	60		
)emocratic People's Republic of Korea									
India	180	100		2009	other	80	40		2009

	Ozone (O3) µg/m3				Nitrogen dioxide (NO2) µg/m3				
	O3 1 hour	O3 8-hr daily max	other	year set, revised, or published	NO2 1-h	NO2 24 hours	NO2 1 year	NO2 other	year set, revised, or published
Indonesia	235	other		2010	other	150	100		2010
Maldives	N/A	N/A			N/A	N/A	N/A		
Myanmar	N/A	N/A		2014	N/A	N/A	N/A		2014
Nepal	N/A	N/A		2003	other	80	40		2003
Sri Lanka	200	other		2008	250	100	N/A	150 (8 hr)	2008
Thailand	200	140		2010	other	320	57		2010
Timor-Leste	N/A	N/A			N/A	N/A	N/A		
**Western pacific**									
Australia	200	170		2008	230	other	60		2008
Brunei Darussalam	N/A	N/A			N/A	N/A	N/A		
Cambodia	200	other		2000	300	100	N/A		2000
China	200	160	city region with diff. values	2014	200	80	40	city region with diff. values	2014
Cook Islands									
Fiji	other	100			200	other	40		
Japan	118	other		!	other	113	N/A		!
Kiribati	N/A	N/A			N/A	N/A	N/A		
Lao People's Democratic Republic	N/A	N/A			N/A	N/A	N/A		
Malaysia	200	120		2015	320	75	N/A		2015
Marshall Islands	N/A	N/A			N/A	N/A	N/A		
Micronesia (Federated States of)	N/A	N/A			N/A	N/A	N/A		
Mongolia	N/A	100		2007	other	40	30	85 (20 mins)	2007
Nauru	N/A	N/A			N/A	N/A	N/A		
New Zealand	150	other		2011	200	100	N/A		2011
Niue	N/A	N/A			N/A	N/A	N/A		
Palau	N/A	N/A			N/A	N/A	N/A		
Papua New Guinea	N/A	N/A		2010	N/A	N/A	N/A		2010
Philippines	140	60		2014	other	150	N/A		2014
Republic of Korea	200	120		2013	190	115	57		2013
Samoa	N/A	N/A			N/A	N/A	N/A		
Singapore	other	147	lower targets by 2020	2014	N/A	N/A	100	lower targets by 2020	2014
Solomon Islands	N/A	N/A			N/A	N/A	N/A		
Tonga	N/A	N/A		2015	N/A	N/A	N/A		2015
Tuvalu	N/A	N/A			N/A	N/A	N/A		
Vanuatu (Palestine)									

	Sulphur dioxide (SO2) µg/m3					
	SO2 10 minutes	SO2 1 hour	SO2 24 hours	SO2 1 year	other	year set, revised, or published
**WHO AQGs**	500		20			2006
**WHO REGIONS AQSs**						
**European Region**						
**EU Directive 2008/50/EC**	other	350	125	N/A		2008
Austria	other	200	120	N/A	20 (y) winter mean vegetation	2010
Belgium	other	350	125	N/A		!
Bulgaria	N/A	N/A	N/A	N/A		2011
Croatia	other	350	125	other	20 (y) winter mean vegetation	2010/2014
Cyprus	other	350	125	N/A		
Czech Republic	other	350	125	N/A		
Denmark	other	350	125	N/A		2010
Estonia	other	350	125	N/A		
Finland	other	350	125	N/A		2011
France	other	350	125	other	20 (y) winter mean vegetation	2012
Germany	other	350	125	50		2002
Greece	other	350	125	N/A		
Hungary	other	350	125	N/A		
Ireland	other	350	125	N/A		2011
Italy	other	350	125	other	20 (1y) vegetation	2010
Latvia	other	350	125	N/A		
Lithuania	other	350	125	other	20 (1y) vegetation	2005
Luxemburg	other	350	125	N/A		2011
Malta	other	350	125	N/A		2015
The Netherlands	other	350	125	N/A		<2006
Poland	other	350	125	N/A		2012
Portugal	other	350	125	N/A		
Romania	other	350	125	N/A		
Slovakia	other	350	125	N/A		
Slovenia	other	350	125	N/A		
Spain	other	350	125	other	20 winter (1y)	2011
Sweden	other	200	100	20	annual in order to protect vegetation	2010
United Kingdom	other	350	125	other	20 (y) winter mean vegetation	2010

	Sulphur dioxide (SO2) µg/m3					
	SO2 10 minutes	SO2 1 hour	SO2 24 hours	SO2 1 year	other	year set, revised, or published
Albania	N/A	N/A	N/A	50		2003
Andorra	other	350	125	20		2009
Armenia	N/A	N/A	N/A	other	1 time 0.5 mg/m3 MPC 50 (1y?)	2006
Azerbaijan	N/A	N/A	other	N/A	MAC 50	2011
Belarus	N/A	N/A	N/A	N/A		2010
Bosnia and Herzegovina	other	350	125	50	Winter vegetation protection 20	2012
Georgia						
Iceland	other	350	125	20		2002
Israel	other	350	50	20		2014
Kazakhstan	N/A	N/A	125	N/A		2012
Kyrgyzstan	N/A	N/A	50	N/A	MAC 500	!
Monaco						
Montenegro	other	350	110	N/A		2015
Norway	other	350	125	20		2007
Republic of Moldova	N/A	N/A	N/A	N/A		
Russian Federation	N/A	N/A	50	N/A	500 (20min)	!
San Marino	N/A	N/A	N/A	N/A		2012
Serbia	other	350	125	50	winter 20	2009
Switzerland	N/A	N/A	100	30		2015
Tajikistan	N/A	N/A	other	other	MAC 50	2010
rmer Yugoslav Republic of Macedonia	other	350	125	20		2011
Turkey	other	500	250	150		2008
Turkmenistan	N/A	N/A	50	N/A	MAC 500	1996
Ukraine	N/A	N/A	N/A	other	MAC 50	
Uzbekistan	N/A	N/A	200	50		2010
**Region of the Americas**						
Antigua and Barbuda	N/A	N/A	N/A	N/A		
Argentina	N/A	N/A	N/A	N/A	(1 month), city region with diff. AQS	2013
Bahamas	other	350	125	other	20 (1y) winter	
Barbados	N/A	N/A	N/A	N/A		2000
Belize	N/A	N/A	N/A	N/A		2003
Bolivia (Plurinational State of)	other	80	365	N/A	city region with diff. 10min 24h AQS	2013
Brazil	other	80	365	N/A		2013
Canada	N/A	N/A	N/A	N/A	city region with diff. 10min AQS	2013

	Sulphur dioxide (SO2) µg/m3					
	SO2 10 minutes	SO2 1 hour	SO2 24 hours	SO2 1 year	other	year set, revised, or published
Chile	other	80	250	N/A		2013
Colombia	other	80	250	N/A		2013
Costa Rica	other	80	365	N/A		2013
Cuba	N/A	N/A	N/A	N/A		2013
Dominica	N/A	N/A	N/A	N/A		2013
Dominican Republic	other	450	150	100		2003
Ecuador	other	80	350	N/A		2013
El Salvador	other	80	365	N/A		2013
Grenada						
Guatemala	N/A	N/A	N/A	N/A		
Guyana	N/A	N/A	N/A	N/A		2000
Haiti	N/A	N/A	N/A	N/A		
Honduras	N/A	N/A	N/A	N/A		
Jamaica	other	700	365	80		2013
Mexico	other	524	288	66		2014
Nicaragua	N/A	N/A	365	80		2013
Panama	N/A	other	365	80		2013
Paraguay	N/A	N/A	20	N/A		2015
Peru	N/A	N/A	20	N/A		2013
Saint Kitts and Nevis	N/A	N/A	N/A	N/A		
Saint Lucia	N/A	N/A	N/A	N/A		2005
Saint Vincent and the Grenadines						
Suriname	N/A	N/A	N/A	N/A		
Trinidad and Tobago	500	other	125	50		2014
United States of America	other	196	N/A	N/A	), city region with diff. values 1h 24h	2016
Uruguay	N/A	N/A	N/A	N/A		
Venezuela (Bolivarian Republic of)	N/A	N/A	365	80		2013
**African Region**						
Algeria	N/A	other	other	N/A	350 (?h)	2006
Angola	N/A	N/A	N/A	N/A		
Benin	other	1300	200	80		2001
Botswana	N/A	N/A	300	80	160 (1 month)	2000
Burkina Faso	other	200	N/A	N/A	200-300 1h	2000
Burundi	N/A	N/A	N/A	N/A		

	Sulphur dioxide (SO2) µg/m3					
	SO2 10 minutes	SO2 1 hour	SO2 24 hours	SO2 1 year	other	year set, revised, or published
Cameroon	500	other	20	N/A		
Cabo Verde						
Central African Republic	N/A	N/A	N/A	N/A		
Chad	N/A	N/A	N/A	N/A		2016
Comoros						
Congo	N/A	N/A	N/A	N/A		
Côte d'Ivoire	N/A	N/A	N/A	N/A		2015
Democratic Republic of the Congo	N/A	N/A	N/A	N/A		
Equatorial Guinea						
Eritrea	N/A	N/A	N/A	N/A		
Ethiopia	N/A	N/A	N/A	N/A		
Gabon	N/A	N/A	N/A	N/A		
Gambia	N/A	N/A	125	50		
Ghana	other	100	N/A	80		2016
Guinea	N/A	N/A	N/A	N/A		
Guinea-Bissau						
Kenya	500	other	80	60		2014
Lesotho	N/A	N/A	N/A	N/A		
Liberia	N/A	N/A	N/A	N/A		
Madagascar	N/A	N/A	N/A	N/A		
Malawi	other	230	210	50		2005
Mali	N/A	N/A	N/A	N/A		
Mauritania						
Mauritius	other	325	186	45		1998
Mozambique	other	800	365	80		2005
Namibia	N/A	N/A	N/A	N/A		
Niger						
Nigeria	N/A	N/A	N/A	N/A		
Rwanda	N/A	N/A	N/A	N/A		2014
Sao Tome and Principe						
Senegal	N/A	N/A	125	50		2003
Seychelles	N/A	N/A	N/A	N/A		
Sierra Leone						
South Africa	500	350	125	50		2009

	Sulphur dioxide (SO2) µg/m3					
	SO2 10 minutes	SO2 1 hour	SO2 24 hours	SO2 1 year	other	year set, revised, or published
South Sudan						
Swaziland	N/A	N/A	125	50		2012
Togo	N/A	N/A	N/A	N/A		
Uganda	N/A	N/A	N/A	N/A		
United Republic of Tanzania	500	other	100	40	to 60 (y)	2007
Zambia	500	350	125	other	50 (6 months)	1996
Zimbabwe	N/A	N/A	N/A	N/A		
**Eastern Mediterranean Region**						
Afghanistan	500	other	20	N/A		2010
Bahrain						
Djibouti						
Egypt	other	350	150	60		1994
Iran (Islamic Republic of)	N/A	N/A	100	20		2011
Iraq	N/A	N/A	N/A	N/A		
Jordan	N/A	N/A	N/A	N/A		!
Kuwait	other	75	20	N/A		!
Lebanon	other	350	120	80		!
Libya						
Morocco	N/A	N/A	125	N/A	20 (y) ecosystem protection	2009
Oman						
Pakistan	N/A	N/A	120	80		2010
Qatar						
Saudi Arabia	other	730	365	80		!
Somalia						
Sudan						
Syrian Arab Republic	other	350	125	N/A		2011
Tunisia						
United Arab Emirates						
Yemen						
**South-East Asia**						
Bangladesh	N/A	N/A	365	80		2005
Bhutan	N/A	N/A	80	60		
Democratic People's Republic of Korea						
India	N/A	N/A	80	50		2009

	Sulphur dioxide (SO2) µg/m3					
	SO2 10 minutes	SO2 1 hour	SO2 24 hours	SO2 1 year	other	year set, revised, or published
Indonesia	N/A	N/A	365	60		2010
Maldives	N/A	N/A	N/A	N/A		
Myanmar	N/A	N/A	N/A	N/A		2014
Nepal	N/A	N/A	70	50		2003
Sri Lanka	other	200	80	N/A	120 (8 hr)	2008
Thailand	other	780	300	100		2010
Timor-Leste	N/A	N/A	N/A	N/A		
**Western pacific**						
Australia	other	500	200	50		2008
Brunei Darussalam	N/A	N/A	N/A	N/A		
Cambodia	other	500	300	N/A		2000
China	other	500	150	60	city region with diff. values	2014
Cook Islands						
Fiji	500	other	20	N/A		
Japan	other	262	105	N/A		!
Kiribati	N/A	N/A	N/A	N/A		
Lao People's Democratic Republic	N/A	N/A	N/A	N/A		
Malaysia	other	250	105	N/A		2015
Marshall Islands	N/A	N/A	N/A	N/A		
Micronesia (Federated States of)	N/A	N/A	N/A	N/A		
Mongolia	500	other	20	10	450 (20 mins)	2007
Nauru	N/A	N/A	N/A	N/A		
New Zealand	other	350	120	50		2011
Niue	N/A	N/A	N/A	N/A		
Palau	N/A	N/A	N/A	N/A		
Papua New Guinea	N/A	N/A	N/A	N/A		2010
Philippines	N/A	N/A	180	80		2014
Republic of Korea	other	400	130	50		2013
Samoa	N/A	N/A	N/A	N/A		
Singapore	N/A	N/A	365	80	lower targets by 2020	2014
Solomon Islands	N/A	N/A	N/A	N/A		
Tonga	N/A	N/A	N/A	N/A		2015
Tuvalu	N/A	N/A	N/A	N/A		
Vanuatu (Palestine)						

	Sulphur dioxide (SO2) µg/m3					
	SO2 10 minutes	SO2 1 hour	SO2 24 hours	SO2 1 year	other	year set, revised, or published
Viet Nam	other	350	125	N/A		2013
**OTHER**						
Gibraltar	other	350	125	N/A		2010
St Helena	N/A	N/A	N/A	N/A		2012
Liechtenstein	N/A	N/A	100	30	100 (1/2h values)	2008
Taiwan	N/A	N/A	266	80		2012

	Carbon Monoxide (CO) mg/m3							
	CO 15 min	CO 1 hour	CO 8 hour	CO 24 hours	CO other	year set, revised, or published	Reference No	Comments
**WHO AQGs**	100	35	10	7		2010		
**WHO REGIONS AQSs**								
**European Region**								
**EU Directive 2008/50/EC**	N/A	N/A	7	N/A		2008	[1]	
Austria	N/A	N/A	10	N/A		2010	[2]	
Belgium	N/A	N/A	10	N/A		!	[3]	
Bulgaria	N/A	N/A	N/A	N/A		2011	[4] [5]	Clean ambient air act exists, no values defined, values from airlex
Croatia	N/A	N/A	10	N/A		2010/2014	[6] [7]	
Cyprus	N/A	N/A	10	N/A			[1]	
Czech Republic	N/A	N/A	10	N/A			[8]	
Denmark	N/A	N/A	10	N/A		2010	[9]	
Estonia	N/A	N/A	10	N/A			[10]	
Finland	N/A	N/A	10	N/A		2011	[11]	
France	N/A	N/A	10	N/A		2012	[12]	
Germany	N/A	N/A	N/A	N/A		2002	[13]	
Greece	N/A	N/A	10	N/A			[1]	
Hungary	N/A	N/A	10	N/A			[1]	
Ireland	N/A	N/A	10	N/A		2011	[14]	
Italy	N/A	N/A	10	N/A		2010	[15]	
Latvia	N/A	N/A	10	N/A		2007	[16]	
Lithuania	N/A	N/A	10	N/A		2005	[16]	maybe Date is mistaken for when to be met
Luxemburg	N/A	N/A	10	N/A		2011	[17]	
Malta	N/A	N/A	10	N/A		2015	[16]	
The Netherlands	N/A	N/A	10	N/A		<2006	[18]	
Poland	N/A	N/A	10	N/A		2012	[19]	
Portugal	N/A	N/A	10	N/A			[16]	
Romania	N/A	N/A	10	N/A			[16]	
Slovakia	N/A	N/A	10	N/A			[16]	
Slovenia	N/A	N/A	10	N/A			[16]	
Spain	N/A	N/A	10	N/A		2011	[20]	
Sweden	N/A	N/A	10	N/A		2010	[21]	
United Kingdom	N/A	N/A	10	N/A		2010	[22]	region with diff. AQS (scotland)
Albania	N/A	40	10	2		2003	[23] [24] [16]	
Andorra	N/A	40	10	N/A		2009	[25]	
Armenia	other	N/A	N/A	3	?), MPC 3.0 ug/m3 (1y?)	2006	[26] [27]	
Azerbaijan	N/A	N/A	N/A	3	MAC 3	2011	[28] [16]	
Belarus	N/A	N/A	N/A	N/A			[29]	
Bosnia and Herzegovina	N/A	N/A	10	5	3 (1y)	2012	[30]	
Georgia							[31]	
Iceland	100	30	10	N/A		2002	[32] [33]	
Israel	N/A	N/A	N/A	N/A			[34]	
Kazakhstan	N/A	N/A	N/A	N/A			[35]	
Kyrgyzstan	N/A	N/A	N/A	N/A		!	[36] [37]	
Monaco							[16]	unknown
Montenegro	N/A	N/A	10	N/A		2015	[38] [39]	

	Carbon Monoxide (CO) mg/m3							
	CO 15 min	CO 1 hour	CO 8 hour	CO 24 hours	CO other	year set, revised, or published	Reference No	Comments
Norway	N/A	N/A	10	N/A		2007	[40]	
Republic of Moldova	N/A	N/A	N/A				[16] [41]	
Russian Federation	other	N/A	N/A	3	5 (20 mins)		[42]	
San Marino	N/A	N/A	N/A	N/A		2012	[43]	
Serbia	N/A	N/A	10	5	3 (1 yr)	2009	[44]	
Switzerland	N/A	N/A	N/A	8		2015	[45]	
Tajikistan	N/A	N/A	N/A	N/A		2010	[46] [47] [48]	
rmer Yugoslav Republic of Macedonia	N/A	N/A	10	N/A			[49]	
Turkey	N/A	N/A	10	N/A			[50]	
Turkmenistan	N/A	N/A	N/A	N/A		1996	[51] [52]	
Ukraine	N/A	N/A	N/A	N/A	MAC 3		[53] [54]	
Uzbekistan	N/A	N/A	N/A	N/A			[55] [53]	
**Region of the Americas**								
Antigua and Barbuda	N/A	N/A	N/A	N/A			[56]	
Argentina	N/A	58	12	N/A	ty region with diff. values		[57]	
Bahamas	N/A	N/A	N/A	N/A			[7]	
Barbados	N/A	N/A	N/A	N/A		2000	[58] [16]	
Belize	N/A	N/A	N/A	N/A		2003	[59] [16]	
Bolivia (Plurinational State of)	N/A	40	10	N/A	ty region with diff. values		[56]	
Brazil	N/A	40	10	N/A			[56]	
Canada	N/A	N/A	N/A	N/A			[60] [61]	
Chile	N/A	30	10	N/A			[56]	
Colombia	N/A	40	10	N/A			[56]	
Costa Rica	N/A	40	10	N/A			[56]	
Cuba	N/A	N/A	N/A	N/A			[56]	
Dominica	N/A	N/A	N/A	N/A			[16] [62]	
Dominican Republic	N/A	40	10	N/A		2003	[63]	
Ecuador	N/A	40	10	N/A			[56]	
El Salvador	N/A	40	10	N/A			[56]	
Grenada							[16]	unclear as of 2005, unknown
Guatemala	N/A	N/A	N/A	N/A			[56]	
Guyana	N/A	N/A	N/A	N/A		2000	[16]	
Haiti	N/A	N/A	N/A	N/A			[56]	
Honduras	N/A	N/A	N/A	N/A			[16] [56]	
Jamaica	N/A	40	10	N/A			[56]	
Mexico	N/A	N/A	N/A	N/A			[64]	
Nicaragua	N/A	40	10	N/A			[56]	
Panama	N/A	30	10	N/A			[56]	
Paraguay	N/A	N/A	10	N/A			[65] [66]	
Peru	N/A	30	10	N/A			[67]	
Saint Kitts and Nevis	N/A	N/A	N/A	N/A			[16]	
Saint Lucia	N/A	N/A	N/A	N/A		2005	[16]	
Saint Vincent and the Grenadines							[16]	unknown
Suriname	N/A	N/A	N/A	N/A			[16]	
Trinidad and Tobago	100	30	10	N/A	60 (30 mins)	2014	[68]	
United States of America	N/A	43	11	N/A			[69] [70]	

	Carbon Monoxide (CO) mg/m3							
	CO 15 min	CO 1 hour	CO 8 hour	CO 24 hours	CO other	year set, revised, or published	Reference No	Comments
Uruguay	N/A	N/A	N/A	N/A			[16] [56]	
Venezuela (Bolivarian Republic of)	N/A	35	10	N/A			[56]	
**African Region**								
Algeria	N/A	N/A	N/A	N/A		2006	[71]	averaging times not given 24h assumed
Angola	N/A	N/A	N/A	N/A			[16]	
Benin	N/A	40	10	N/A		2001	[7] [72]	Standards have to be promulgated
Botswana	N/A	40	10	N/A		2000	[72] [16]	The emission and AQS setting stipulated in Atmospheric Pollution Prevention A
Burkina Faso	N/A	30	N/A	N/A			[72] [73]	Standards have been adopted
Burundi	N/A	N/A	N/A	N/A			[72] [16]	Standards have not been introduced
Cameroon	100	35	10	7			[72] [16]	Have to be promulgated
Cabo Verde							[16]	unknown
Central African Republic	N/A	N/A	N/A	N/A			[16]	
Chad	N/A	N/A	N/A	N/A		2016	[74]	
Comoros							[16]	unknown
Congo	N/A	N/A	N/A	N/A			[16] [72]	Not yet promulgated as of 2006
Côte d'Ivoire	N/A	N/A	N/A	N/A		20151	[16]	in the process of setting standards
Democratic Republic of the Congo	N/A	N/A	N/A	N/A			[72]	Standards have to be promulgated as of 2006
Equatorial Guinea							[16]	unknown
Eritrea	N/A	N/A	N/A	N/A			[16]	
Ethiopia	N/A	N/A	N/A	N/A			[72]	Do not appear to be promulgated. US EPA standards are used.
Gabon	N/A	N/A	N/A	N/A			[72]	Have not been set
Gambia	N/A	N/A	N/A	N/A			[7]	
Ghana	N/A	N/A	N/A	N/A		2016	[7] [16]	
Guinea	N/A	N/A	N/A	N/A			[72]	Standards have to be promulgated as of 2006
Guinea-Bissau							[16]	unknown
Kenya	N/A	N/A	2	N/A		2014	[75]	Standards are "Ambient Air Quality Tolerance Limits"
Lesotho	N/A	N/A	N/A	N/A			[16]	
Liberia	N/A	N/A	N/A	N/A			[72]	Standards have to be promulgated
Madagascar	N/A	N/A	N/A	N/A			[72]	Standards are not yet promulgated (2006)
Malawi	N/A	40	10	N/A		2005	[76]	
Mali	N/A	N/A	N/A	N/A			[16]	Standards have to be promulgated
Mauritania							[16] [77]	unknown
Mauritius	N/A	23	9	N/A			[72] [78] [77]	Standards were set in 1998 and are under review
Mozambique	N/A	40	10	N/A		2005	[79]	
Namibia	N/A	N/A	N/A	N/A			[16]	
Niger							[16]	unknown
Nigeria	N/A	N/A	N/A	N/A			[72] [80]	no mention on air quaility standards but noise found
Rwanda	N/A	4	2	N/A		2014	[16] [7]	
Sao Tome and Principe							[16]	unknown
Senegal	N/A	N/A	N/A	30	0.5 (1 yr)	2003	[81]	
Seychelles	N/A	N/A	N/A	N/A			[16]	
Sierra Leone							[16]	unknown
South Africa	N/A	30	10	N/A		2009	[82] [83]	
South Sudan							[16]	unknown
Swaziland	N/A	N/A	N/A	N/A			[77]	Objectives
Togo	N/A	N/A	N/A	N/A			[72]	AQM is at an initial (very early) stage, Standards are not promulgated

	Carbon Monoxide (CO) mg/m3							
	CO 15 min	CO 1 hour	CO 8 hour	CO 24 hours	CO other	year set, revised, or published	Reference No	Comments
Uganda	N/A	N/A	N/A	N/A			[16]	2006:Standards developed, in stage of becoming law (enforcement weak)
United Republic of Tanzania	100	30	10	N/A	60 (30 mins)		[84]	
Zambia	100	30	10	N/A	nonths), 1.0 (12 months)		[85]	
Zimbabwe	N/A	N/A	N/A	N/A			[16]	Standards are not yet promulgated as of 2006
**Eastern Mediterranean Region**								
Afghanistan	100	35	10	7		2010	[16] [86]	
Bahrain							[16]	unknown
Djibouti							[16]	unknown
Egypt	N/A	30	10	N/A		1994	[87]	
Iran (Islamic Republic of)	N/A	40	10	N/A		2011	[88]	
Iraq	N/A	N/A	N/A	N/A			[89] [16]	
Jordan	N/A	N/A	N/A	N/A			[7]	
Kuwait	N/A	N/A	10	N/A		!	[7]	Guideline values
Lebanon	N/A	30	10	N/A		!	[7]	
Libya							[72]	UNEP: Standards are not yet promulgated, unknown
Morocco	N/A	N/A	N/A	N/A		2009	[90]	
Oman							[16]	
Pakistan	N/A	10	5	N/A		2010	[91]	effective by 1.1.2013
Qatar						2002	[16]	said to be higher than WHO, no specific data available from UNEP
Saudi Arabia	N/A	40	10	N/A		!	[16] [7]	
Somalia							[16]	unknown
Sudan							[16]	there are AQS according to UNEP but unspecified by focal point
Syrian Arab Republic	N/A	N/A	N/A	N/A		2011	[16]	UNEP: Ambient air quality standards has updated in 2011
Tunisia							[16]	
United Arab Emirates							[16]	unknown
Yemen							[16]	unknown
**South-East Asia**								
Bangladesh	N/A	40	10	N/A		2005	[92]	
Bhutan	N/A	4	2	N/A			[93] [16] [7]	Maximum permissible limits
Democratic People's Republic of Korea								unknown
India	N/A	4	2	N/A		2009	[94]	
Indonesia	N/A	30	N/A	N/A		2010	[95]	
Maldives	N/A	N/A	N/A	N/A			[16]	
Myanmar	N/A	N/A	N/A	N/A		2014	[96]	first steps taken to develop AQS
Nepal	100	N/A	10	N/A		2003	[97]	
Sri Lanka	N/A	30	10	N/A		2008	[98]	
Thailand	N/A	30	9	N/A		2010	[99]	
Timor-Leste	N/A	N/A	N/A	N/A			[16]	
**Western pacific**								
Australia	N/A	N/A	11	N/A		2008	[100] [101]	
Brunei Darussalam	N/A	N/A	N/A	N/A			[16]	
Cambodia	N/A	40	20	N/A		2000	[102]	
China	N/A	10	N/A	4	ty region with diff. values	2014	[103] [104]	Level II, Hong Kong and Macau with diff. values
Cook Islands							[16]	unknown
Fiji	100	35	10	7			[16]	
Japan	N/A	12.5	25	N/A		!	[105]	

	Carbon Monoxide (CO) mg/m3							
	CO 15 min	CO 1 hour	CO 8 hour	CO 24 hours	CO other	year set, revised, or published	Reference No	Comments
Kiribati	N/A	N/A	N/A	N/A			[16]	
Lao People's Democratic Republic	N/A	N/A	N/A	N/A			[16]	
Malaysia	N/A	35	10	N/A		2015	[106]	
Marshall Islands	N/A	N/A	N/A	N/A			[16]	
Micronesia (Federated States of)	N/A	N/A	N/A	N/A			[16]	
Mongolia	other	30	10	N/A	60 (30 mins)	2007	[107]	
Nauru	N/A	N/A	N/A	N/A			[16]	
New Zealand	N/A	N/A	10	N/A		2011	[108]	
Niue	N/A	N/A	N/A	N/A			[16]	
Palau	N/A	N/A	N/A	N/A			[16]	
Papua New Guinea	N/A	N/A	N/A	N/A		2010	[109]	
Philippines	N/A	35	10	N/A		2014	[110]	
Republic of Korea	N/A	31.25	11.25	N/A		2013	[111]	
Samoa	N/A	N/A	N/A	N/A			[16]	
Singapore	N/A	40	10	N/A	lower targets by 2020	2014	[95] [112]	
Solomon Islands	N/A	N/A	N/A	N/A			[16]	
Tonga	N/A	N/A	N/A	N/A		2015	[16]	
Tuvalu	N/A	N/A	N/A	N/A			[16]	
Vanuatu (Palestine)								unknown
Viet Nam	N/A	30	10	N/A		2013	[113]	
**OTHER**								
Gibraltar	N/A	N/A	7	N/A		2010	[114]	
St Helena	N/A	N/A	N/A	N/A		2012	[115]	
Liechtenstein	N/A	N/A	10	8		2008	[116]	
Taiwan	N/A	40.8	10.5	N/A		2012	[117]	

Online Resource 2. References to Online Resource 1.

1 European Parliament, *Directive 2008/50/EC of the European Parliament and of the Council of 21 May 2008 on ambient air quality and clear air for Europe.* . 2008, Official Journal of the European Union.

2 Österreich: Umwelt Bundesamt. Limit, target and threshold values. 2016 23.06.2016]; Available from: http://www.umweltbundesamt.at/umweltsituation/luft/luftguete_aktuell/grenzwerte/.

3 Belgian Interregional Environment Agency, *ANNUAL REPORT Air Quality in Belgium 2011* . 2012, Belgian Interregional Environment Agency.

4 Bulgaria: Ministry of the Environment, *Clean Ambient Air Act* , M.o.t. Environment, Editor. 2011.

5 UNECE, *Environmental Performance Reviews: Bulgaria, 2nd Review* . 2000, United Nations Economic Commission for Europe.

6 Croatian Environment Agency, *Regulations* . 2015.

7 Universidade de Aveiro: Instituto do Ambiente e Desenvolvimento. Airlex - Worldwide Air Quality Legislation. 2013 6.5.2013 [cited 2016 16.6.2016]; Available from: http://airlex.web.ua.pt/.

8 Czech Hydrometeorological Institute, *AQIS emission limits* , C.H. Institute, Editor. 2012.

9 Denmark, *Order on the assessment and management of air quality* 2006.

10 European Environment Agency, *Air pollution fact sheet 2014: Estonia.* 2014.

11 Environmental Administration of Finland, *Air pollution control* , E. Administration, Editor. 2015.

12 French Republic: Ministry of the environment, e., and sea,, *Air quality standards* , d.l.É.e.d.l.M.M.o.t.e. Ministère de l'Environnement, energy, and sea], Editor. 2012 (updated 2015).

13 German Federal Ministry for the Environment, *Erste Allgemeine Verwaltungsvorschrift zum Bundes-Immissionsschutzgesetz (Technische Anleitung zur Reinhaltung der Luft-TA Luft) - First General Administrative Provision on the Federal Control Act* , B. (Deutschland), Editor. 2002.

14 Irish Government, *Air Quality Standards and Regulations 2011* . Iris Oifigiúil.

15 GAZZETTA UFFICIALE DELLA REPUBBLICA ITALIANA, *LEGGI ED ALTRI ATTI NORMATIVI* . 2010: GAZZETTA UFFICIALE DELLA REPUBBLICA ITALIANA Serie generale - n. 23.

16 United Nations Environment Programme, *Air quality policy catalogue* . 2015.

17 The Government of Luxemburg, *Air quality for Luxemburg* , M.f.L.-t.D.a. Infrastructures, Editor. 2011.

18 Rijkswaterstaat: Ministerie van Infrastrtuur en Milieu (The Netherlands). Grenswaarden en andere luchtkwaliteitsnormen. 2016 [cited 2016 8.7.2016]; Available from: http://www.infomil.nl/onderwerpen/klimaat-lucht/luchtkwaliteit/regelgeving/wet-milieubeheer/beoordelen/grenswaarden/.

19 Republic of Poland: Department of the Environment, *On the levels of certain substances in the air* , D.o.t. Environment, Editor. 2012.

20 Spain: Ministry of the Presidency, *General Provision: Real Decreto 102/2011, de 28 de enero, relativo a la mejora de la calidad del aire.* , M.o.t. President, Editor. 2011.

21 Swedish Environmental Protection Agency, *Swedish Code of Statutes: Air quality ordinance; SFS 2010:477* S.E.P. Agency, Editor. 2010.

22 United Kingdom: Department for Environment, F.a.R.A., *Air quality strategy for England, Scotland, Wales and Northern Ireland* , F.a.R.A. Department for Environment, Editor. 2007.

23 European Environment Agency, *Air pollution - State and impacts (Albania)* . 2015, European Environment Agency.

24 E-on, *Health Impact Assessment: Trans Adriatic Pipeline: ESIA Albania Annex 8.1 – Impact Assessment Data* . 2010.

25 Government of Andorra, *Butlletí Oficial del Principat d'Andorra: Reglament de control de la contaminació atmosfèrica* . 2009.

26 Petikyan, A., *Air quality standards for Armenia* , M.K. Joss, Editor. 2015.

27 Armenia: Environmental Inspection of the Ministry of Nature Protection, *Air quality* , E.I.M.C. Armenia, Editor. 2002.

28 Europe, U.N.E.C.f., *Environmental Performance Review: Azerbaijan, 2nd Review.* 2011.

29 UNECE, *Environmental Performance Review: Belarus, 3rd Review* . 2014.

30 Bosnia & Herzegovenia: Ministry of the Environment and Tourism, *Rules on the method of monitoring air quality and defining the types of pollutants, limit values and other standards for air quality* , M.o.t.E.a. Tourism, Editor. 2012.

31 UNECE, *Environmental Performance Review: Georgia, 3rd review* . 2016, United Nations Economic Commission for Europe.

32 Einarsson, E.e.a., *Hreint loft, betri heilsa - Umfjöllun um loftgæði og heilsufar á Íslandi ásamt tillögum til úrbóta* , E.a.N.R. Ministry, Editor. 2013, Iceland: Environment and Natural Resources Ministry: Reykjavik, Iceland.
33 Skúladóttir, B.T., A.; Larssen, S.; Bjarnason, G.; Þórðarson, H., *Method for determining the composition of airborne particle pollution.* Nordtest, 2003.
34 Dobnov, J., *Monitoring and modeling air polluton and its health effects: situation in Israel* . 2014.
35 Republic of Kazakhstan, *Sanitary-epidemiological requirements for air quality in urban and rural areas, soils, and their security, maintenance of areas of urban and rural settlements, conditions of work with sources of physical factors affecting humans, S 168* . 2012.
36 Kyrgyz Hydromet, *Air pollution monitoring network of Kyrgyzstan* , M.K. Joss, Editor. 2015.
37 UNECE, *Environmental Performance Review: Kyrgyzstan, 2nd review* . 2009, United Nations Economic Commission for Europe.
38 Government of Montenegro, *Regulation: Determination of Types of Pollutants, Limit Values, and Other Air Quality Standards* . 2007.
39 Government of Montenegro, *Law on Air pollution* . 2012.
40 Norwegian Climate and Environment Ministry, *Chapter 7. Local air quality* E. Agency, Editor. 2013.
41 Cojocaru, A., *Air Pollution in the Republic of Moldova: Current Status and Future Prospects* . 2008, Springer Science + Business Media B.V.
42 Ministry of Health of the Russian Federation, h.s.s.d.o.t.R.F., *Maximum Allowable Concentration (MAC) of pollutants in ambient air in populated areas" Hygiene Norms (HG) 2.1.6.1338-03* , M.o.H.o.t.R. Federation, Editor. 2003.
43 Republic of San Marino, *Environmental Code* . 2009.
44 Republic of Serbia, *Regulation: On air quality requirements and monitoring conditions, official gazette of RS no. 1110 and no. 7510* , Ministry of Agriculture and Environmental Protection, Editor. 2009.
45 Schweizerischer Bundesrat, *Luftreinhalte-Verordnung (LRV)* . 1985 (Update 2016).
46 UNECE, *Environmental Performance Review: Tajikistan, 1st review* . 2004, United Nations Economic Commission for Europe: New York and Geneva.
47 Ministry of Transport and Communications for the Asian Development Bank (ADB), *Environmental Impact Assessment: Tajikistan: CAREC Corridor 3 (Dushanbe–Uzbekistan Border) Improvement Project.* 2010.
48 UNECE, *Environmental Performance Reviews: Tajikistan, 2nd Review* , U.N.E.C.f. Europe, Editor. 2012.
49 Republic of Macedonia, *Air quality assessment report* , M.o.E.a.P. Planning, Editor. 2012.
50 Turkish Ministry for the Environment and Forest (Çevre ve Orman Bakanlığı), *Hava kalitesi degerlendirme ve yönetmeligi* , in *Resmî Gazete* . 2008, Çevre ve Orman Bakanlığı: Ankara.
51 State Power Corporation ”Turkmenergo” of the Ministry of Energy of Turkmenistan for the Asian Development Bank, *TKM: Power Sector Efficiency Improvement and Export Project* . 2012.
52 UNECE, *Environmental Performance Review: Turkmenistan, 1st review synopsis* . 2012, United Nations Economic Commission for Europe.
53 World Health Organization, *Health basis for air quality management in Eastern Europe, Caucasus and Central Asia* . 2005.
54 UNECE, *Environmental Performance Review: Ukraine, 2nd review* . 2007, United Nations Economic Commission for Europe.
55 Republic of Uzbekistan, *Hygiene norms on ambient air quality in human settlements of the Republic of Uzbekistan* , C.o.H.S.o.t.R.o. Uzbekistan, Editor. 2010.
56 Clean Air Institute, *Air quality in Latin America: an overview* , C.A. Institute, Editor. 2013.
57 Argentina: Government of Buenos Aires, *National Law 20.284* , P.a.M.A.o.t.C.o.B.A. National Health, Editor. 1973.
58 Global Environmental Outlook - Barbados, *State of the Environment Report 2000: Chapter 8: Atomosphere and Climate* . 2000.
59 Government of Belize, *Subsidiary Laws of Belize: Environmental Protection: Chapter 238: Pollution Regulations* . 2003: Belmopan, Belize.
60 Canada: Environment and Climate Change, *Canadian ambient air quality standards* , E.a.C. Change, Editor. 2015.
61 British Columbia, *Provincial air quality objective: information sheet.* 2016.
62 IEA Clean Coal Centre, *Dominica* . unknown, IEA Clean Coal Centre,.
63 Dominican Republic: State Secretary on Air Quality and Natural Resources, *Ambient Norms on Air Quality and Emissions Control* , S.S.o.A.Q.a.N. Resources, Editor. 2003: Santo Domingo, Dominican Republic.

64 Mexico: Secretary of Health, *Proyecto de norma oficial mexicana proy-nom-025-SSA1-2014, salud ambiental. Valores limite permisibles para la concentracion de particulas suspendidas PM10 y PM2.5 en el aire ambiente y criterios para su evaluacion* , S.o. Health, Editor. 2014.
65 Government of Paraguay, *Permissible Air Quality Standards* , S.o.t. Environment, Editor. 2015: Asuncion, Paraguay.
66 Government of Paraguay, *PARAGUAY ADOPTS AIR QUALITY STANDARDS* , S.o.t.E.o. Paraguay, Editor. 2015.
67 Congress of the Republic of Peru, *Legal norms* . 2001, El Peruano.
68 Republic of Trinidad and Tobago, *The Environmental Management Act, Chap. 35:05: Air Pollution Rules, 2014* . 2015.
69 US Environmental Protection Agency. National Ambient Air Quality Standards Table. 2016 5.7.2016 [cited 2016 3.7.2016]; Available from: https://www.epa.gov/criteria-air-pollutants/naaqs-table.
70 California Air Resource Board, *Ambient air quality standards* , C.A.R. Board, Editor. 2015.
71 LA REPUBLIQUE ALGERIENNE, *Décret définissant les valeurs limites, les seuils d*[illegible]*lerte et les objectifs de qualité de l'air en cas de pollution atmosphérique* . 2006: JOURNAL OFFICIEL DE LA REPUBLIQUE ALGERIENNE N° 01.
72 Schwela, D., *Review of Urban Air Quality in Sub-Saharan Africa Region - Air Quality profile of SSA countries* . 2012, World Bank: Washington, DC. p. 251.
73 Republic of Burkina Faso, *Decree 20010185/PRES/PM/MEE Standards for emissions of air pollutants* . 2000.
74 Kessely, H., *Mail: stanard valeurs pour qualité de l'air au Tchad?* 2016.
75 Kenya, *THE ENVTRONMENTAL MANAGEMENT AND CO-ORDINATION (AIR QUALITY) REGULATIONS* , in *Kenya Gazette Supplement No.41* , Kenya Cabinet Secretary, Editor. 2014, Government Printer: Nairobi.
76 Mapoma, H.W.T.T., Chifundo; Tsakama, Madalitso; Kosamu, Ishmael Bobby Mphangwe, *Air quality assessment of carbon monoxide, nitrogen dioxide and sulfur dioxide levels in Blantyre, Malawi: a statistical approach to a stationary environmental monitoring station.* African Journal of Environmental Science and Technology, 2014. **8**(6): p. 330-343.
77 South African Development Community, *SADC environmental legislation, 3rd edition* , S.A.D. Community, Editor. 2012.
78 Government of Mauritius, *Mauritius Environment Outlook Report 2011* , M.o.E.S. Development, Editor. 2011.
79 Government of the Republic of Mozambique, *Handbook on Environmental Assessment Legislation in the SADC Region* . 2000.
80 Nigeria: Federal Ministry of Environment. Environmental Policies. 2016 30.1.2014 [cited 2016 1.7.2016]; Available from: http://environment.gov.ng/index.php/downloads/3-environmental-policies.
81 DEEC Senegal, *Senegalese Environmental Act: Atmospheric pollution: emission standards, NS 05-062, Octobre 2003* . 2003.
82 South African Bureau of Standards, *South African national standards: ambient air quality - limits for common pollutants* , S.A.B.o. Standards, Editor. 2012, Departement of Environmental Affairs.
83 Republic of South Africa, *Ambient air quality - Limits for common pollutants* , in *SANS 1929 (2011)* . 2011, Republic of South Africa,.
84 Government of Tanzania, *THE ENVIRONMENTAL MANAGEMENT (AIR QUALITY STANDARDS) REGULATIONS* . 2007: Dar es Salaam.
85 Zambia, *Environmental protection and pollution control (amendment) act, Cap 204* .
86 Malikyar, G.M., *Air Quality Management in Afghanistan* , in *Fifth Governmental Meeting on Urban Air Quality in Asia* . 2014, Afghanistan: National Environment Protection Agency,: Sri Lanka.
87 Egypt: Ministry of State for Environmental Affairs Egyptian Environmental Affairs Agency, *Executive Regulations of the Environmental Law no. 4 of Egypt* M.o.S.f.E.A.E.E.A. Agency, Editor. 2009.
88 Iran: Department of the Environment, *Human's Environmental Laws, Regulation Criteria and Standards* , D.o.t. Environment, Editor. 2013: Teheran. p. 339.
89 Iraq: Ministry of the Environment, *The National Enviromental Strategy & Action Plan for Iraq* , M.o.t. Environment, Editor. 2013.
90 UNECE, *Environmental Performance Review: Morocco* . 2014, United Nations Economic Commission for Europe.
91 Government of Pakistan, *National Environmental Quality Standards for Ambient Air* , M.o. Environment, Editor. 2010, Government of Pakistan: Islamabad, Pakistan.
92 Clean Air Initiative for Asian Cities, *Country Synthesis Report, Urban Air Quality Management: Bangladesh.* 2006.
93 Asian Development Bank and the Clean Air Initiative for Asian Cities (CAI-Asia) Center, *Country Synthesis Report on Urban Air Quality Management: Bhutan* . 2006, Asian Development Bank and the Clean Air Initiative for Asian Cities (CAI-Asia) Center.

94 India: Central Pollution Control Board, M.o.E.F., *National Ambient Air Quality Status & Trends 2012.* 2014.
95 Clean Air Initiative for Asian Cities, *Air Quality in Asia: Status and Trends, 2010 edition.* 2010.
96 ASEAN - German Technical Cooperation Clean Air for Smaller Cities in the ASEAN Region, *Myanmar Country Profile: Focus on Cities* , D.O.M.T.H.M.K.P.M.C. Capadocia, Editor. 2014.
97 Nepal: Ministry of Environment, *Annex 7.1 National Ambient Air Quality Standards for Nepal, 2003* 2003.
98 Sri Lanka: Central Environmental Authority, *The Gazete of the Democratic Socialist Republic of Sri Lanka: No. 1562/22.* 2008.
99 Thailand: Ministry of Natural Resources and Environment: Pollution Control Department, *Thai Environmental Regulartions: Air quality and noise standards* , M.o.N.R.a.E.P.C. Department, Editor. 2008.
100 Australia: Department of the Environment, *Air quality standards* , D.o.t. Environment, Editor. 2008.
101 Morawka, L., *Email: Ambient Air Quality Standards for Territories / Islands under the Sovereignty of Australia* . 2016.
102 Cambodia (the Kingdom of), *ANUKRET on The Control of Air Pollution and Noise Disturbance.* 2000.
103 TransportPolicy.net, *China: Air Quality Standards* . 2014.
104 Yang, X., *Email: AQS for Macau and Hong Kong* , M. Kutlar Joss, Editor. 2016.
105 Japan: Ministry of the Environment, *Environmental quality standards in Japan - air quality* , M.o.t. Environment, Editor.
106 Malaysia: Ministry of Environmental Resources & Management, *New Malaysia Ambient Air Quality Standard.* ?
107 Mongolia: Air quality department, *Air quality Technical Requirements (MNS 4585: 2007).* 2007.
108 New Zealand: Ministry for the Environment, *Resource management (national environmental standards for air quality): regulations 2004, reprinted with amendments 17 October 2014* , M.f.t. Environment, Editor. 2011.
109 Esso Highlands Limited, *Papua New Guinea LNG Project: Environmental and Social Management Plan Appendix 2: Air Emissions Management Plan* . 2010.
110 Environmental Management Bureau of the Philippines, *National air quality status report 2010-2011* , D.o.E.a.N. Resources, Editor. 2012: Quezon City, Philippines.
111 Government of Korea, *Air quality standards and air pollution levels* , M.o. Environment, Editor. 2016.
112 Singapore: National Environment Agency. Air Quality and Targets. 2016 26.3.2016 [cited 2016 9.7.2016]; Available from: http://www.nea.gov.sg/anti-pollution-radiation-protection/air-pollution-control/air-quality-and-targets.
113 The Socialist Republic of Vietnam, *National technical regulation on ambient air quality, QCVN 05:2013/BTNMT* . 2013.
114 Environmental Agency for the Government of Gibraltar. Air Quality in Gibraltar: Air Quality Standards. 2016 [cited 2016 8.7.2016]; Available from: http://www.gibraltarairquality.gi/air-quality/.
115 Pelembe, T., *St. Helena: Draft National Environmental Management Plan 2012 - 2022 (Draft)* . 2012, Environmental Management Directorate St. Helena,. p. 30.
116 Liechtenstein, *Luftreinhalteverordnung (LRV) vom 30. September 2008* , in *245* , Liechtenstein, Editor. 2008, Liechtensteinisches Landesgesetzblatt.
117 Taiwan: Environmental Prodection Administration Executive Yuan R.O.C. (Taiwan). Taiwan Air Quality Monitoring Network: Air Quality Standards. 2016 5.7.2016 [cited 2016 11.7.2016]; Available from: http://taqm.epa.gov.tw/taqm/en/b0206.aspx.

Online Resource 3. World map of national air quality standards for annual mean concentration of PM2.5 in relation to WHO air quality guideline values (WHO-AQG) and interim targets.
(*TSP* total suspended particulates)

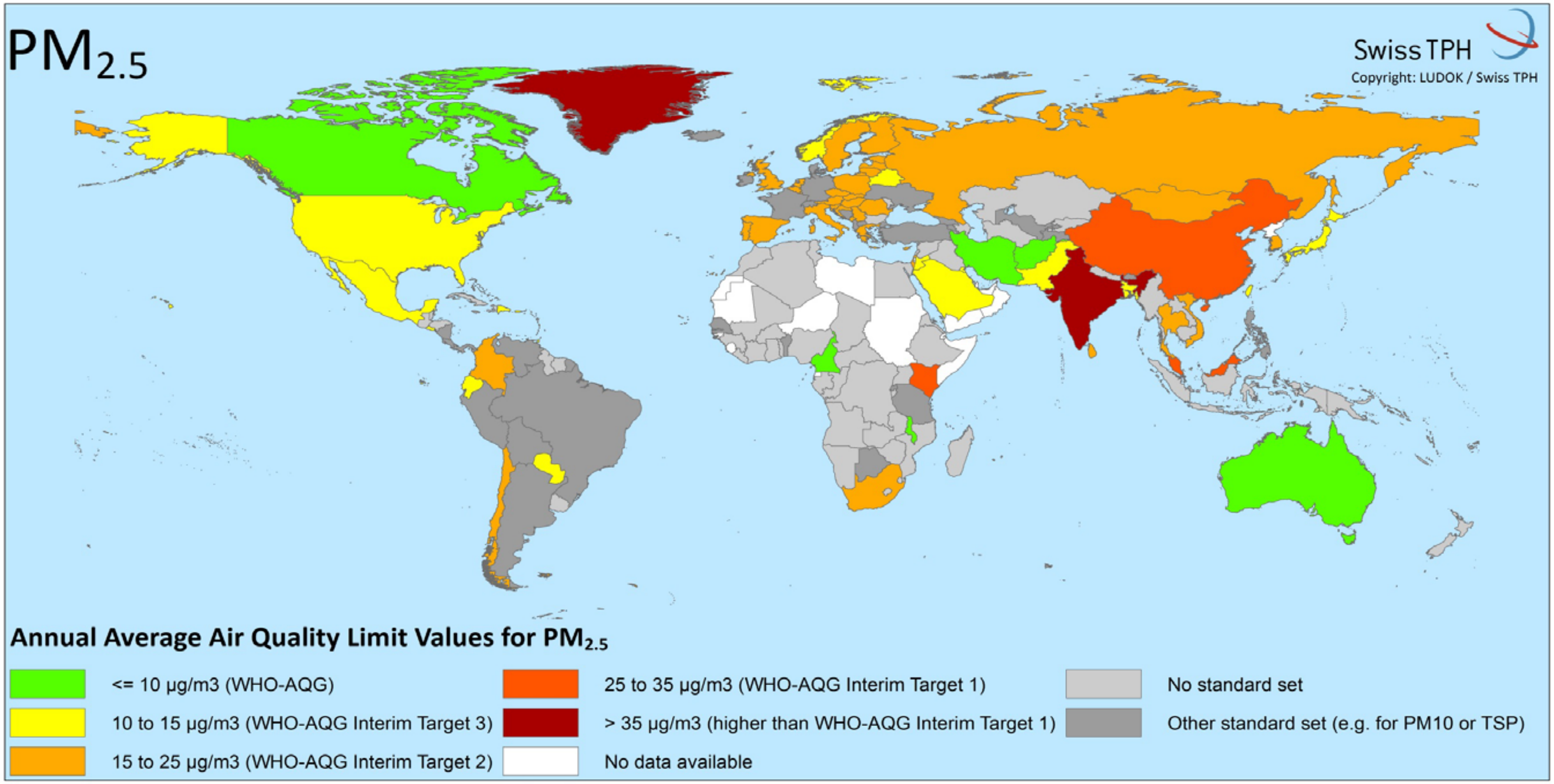

Online Resource 4. World map of national air quality standards for annual mean concentration of SO2 in relation to WHO air quality guideline values (WHO-AQG) and interim targets.

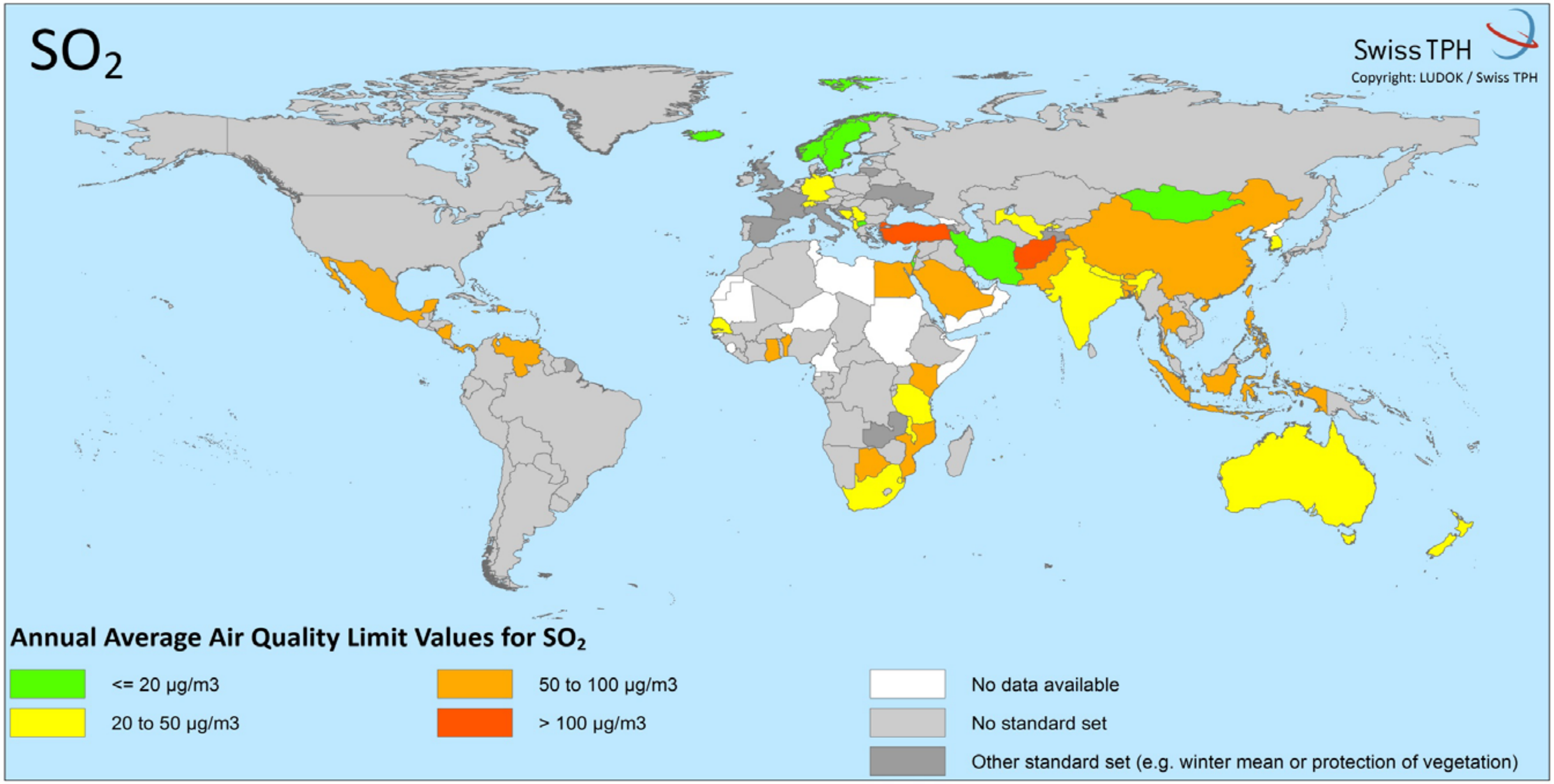

Online Resource 5. World map of national air quality standards for daily mean concentration of PM10 in relation to WHO air quality guideline values (WHO-AQG) and interim targets.
(*TSP* total suspended particulates)

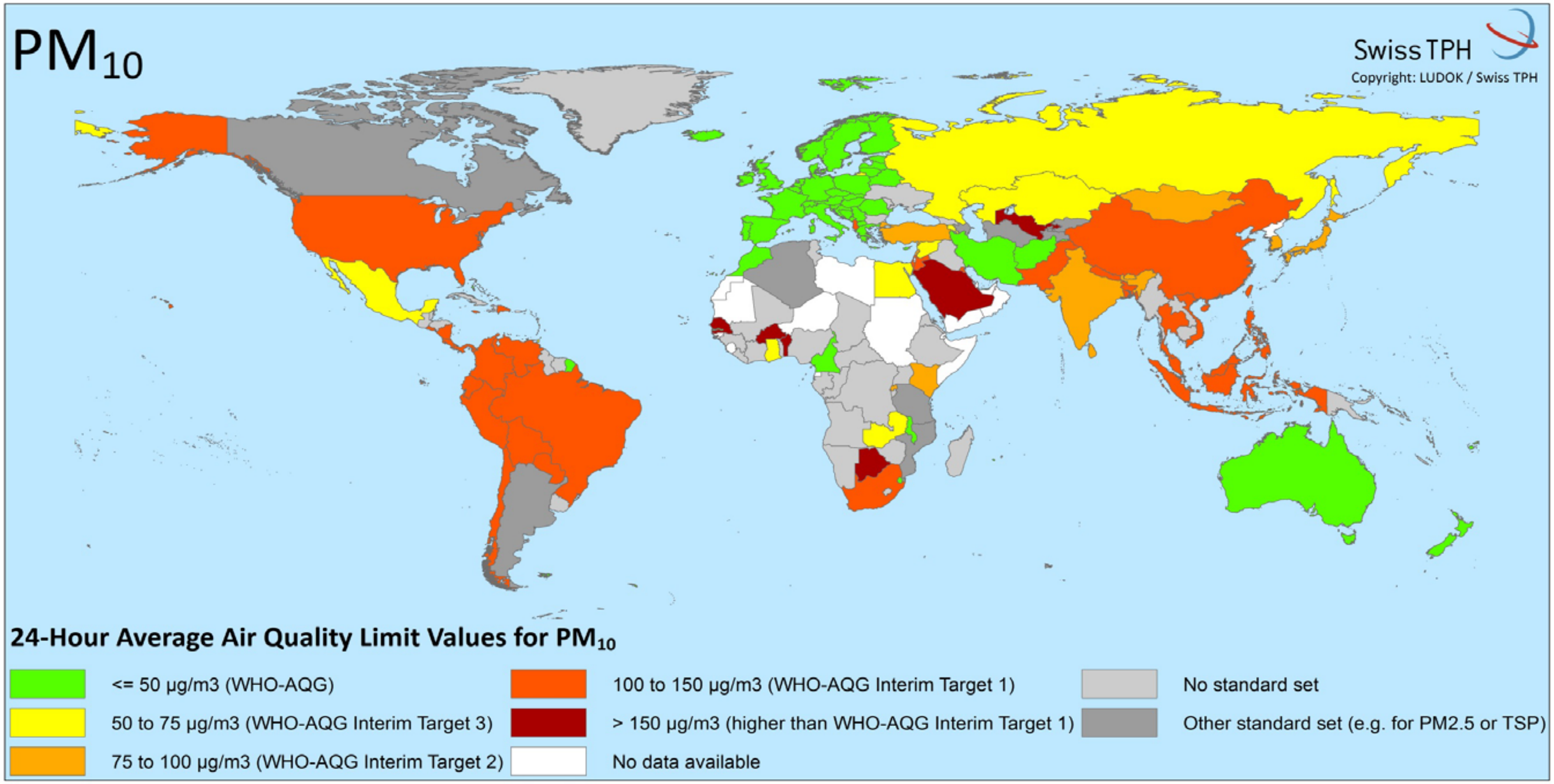

Online Resource 6. World map of national air quality standards for daily mean concentration of PM2.5 in relation to WHO air quality guideline values (WHO-AQG) and interim targets.
(*TSP* total suspended particulates)

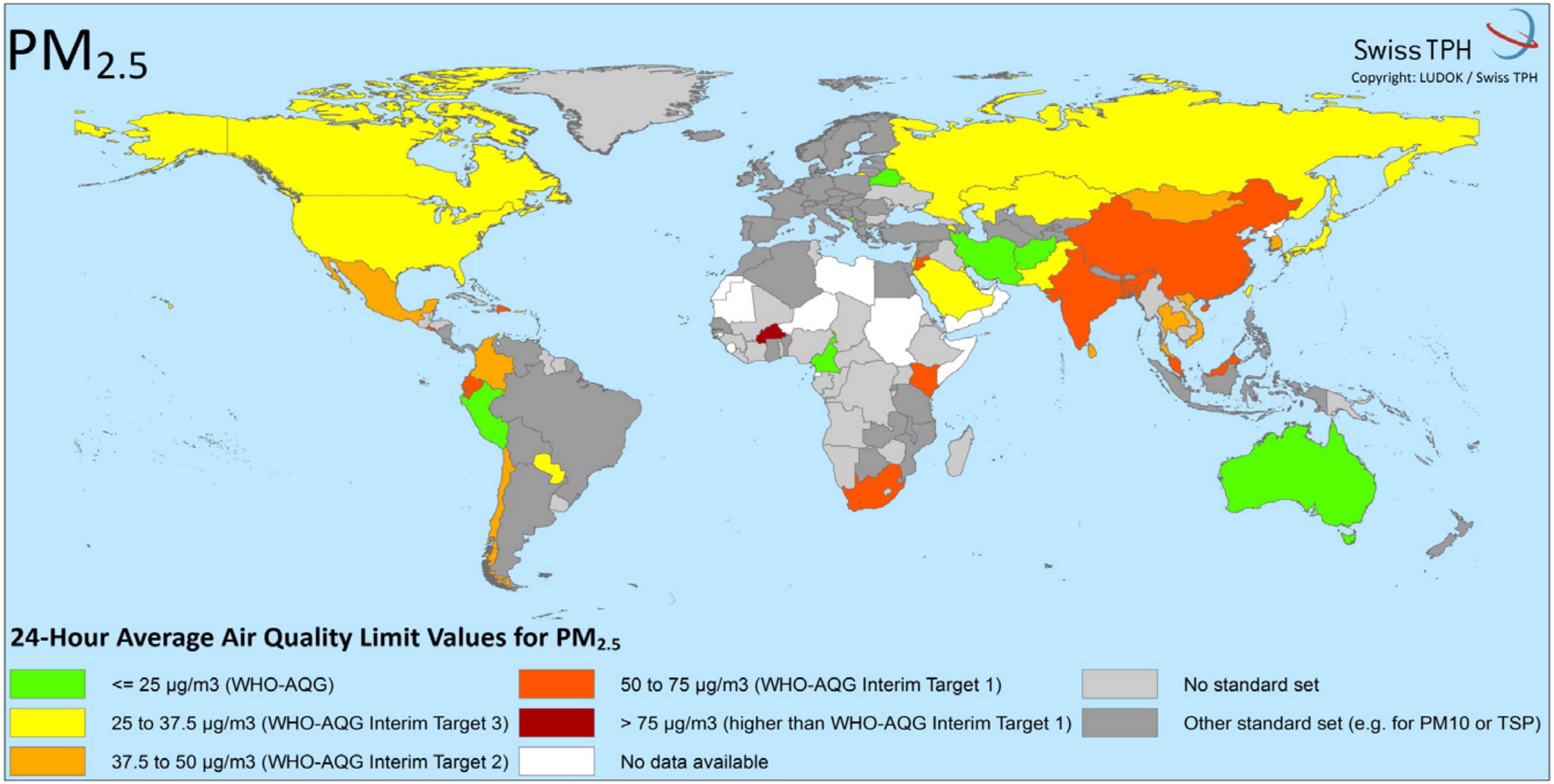

Online Resource 7. World map of national air quality standards for daily mean concentration of SO2 in relation to WHO air quality guideline values (WHO-AQG) and interim targets.
(*MAC* maximum allowable concentration)

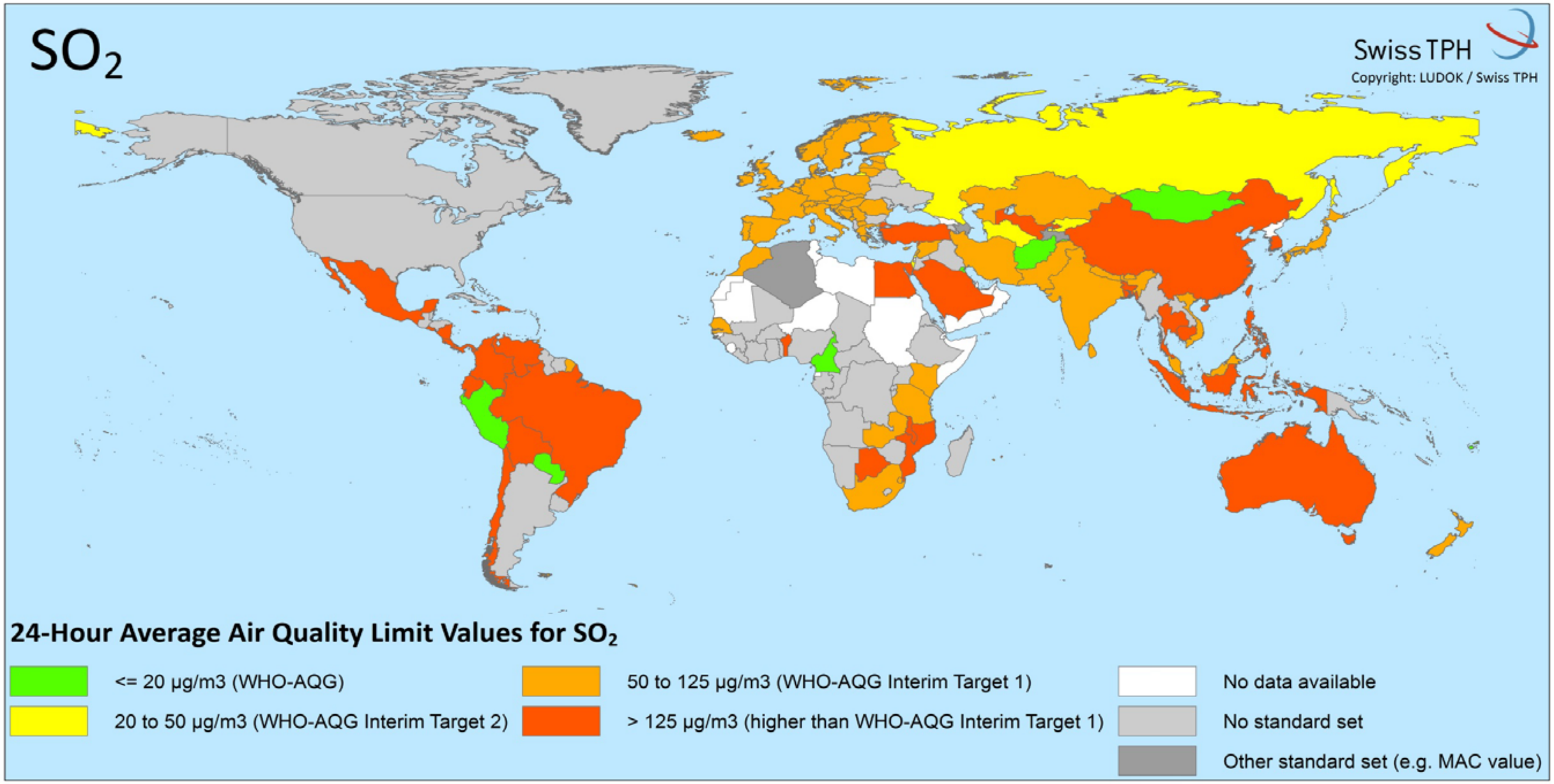

Online Resource 8. World map of national air quality standards for 1-hour average concentration of NO2 in relation to WHO air quality guideline values (WHO-AQG) and interim targets.
(*NOx Oxides of nitrogen: NO and NO2*)

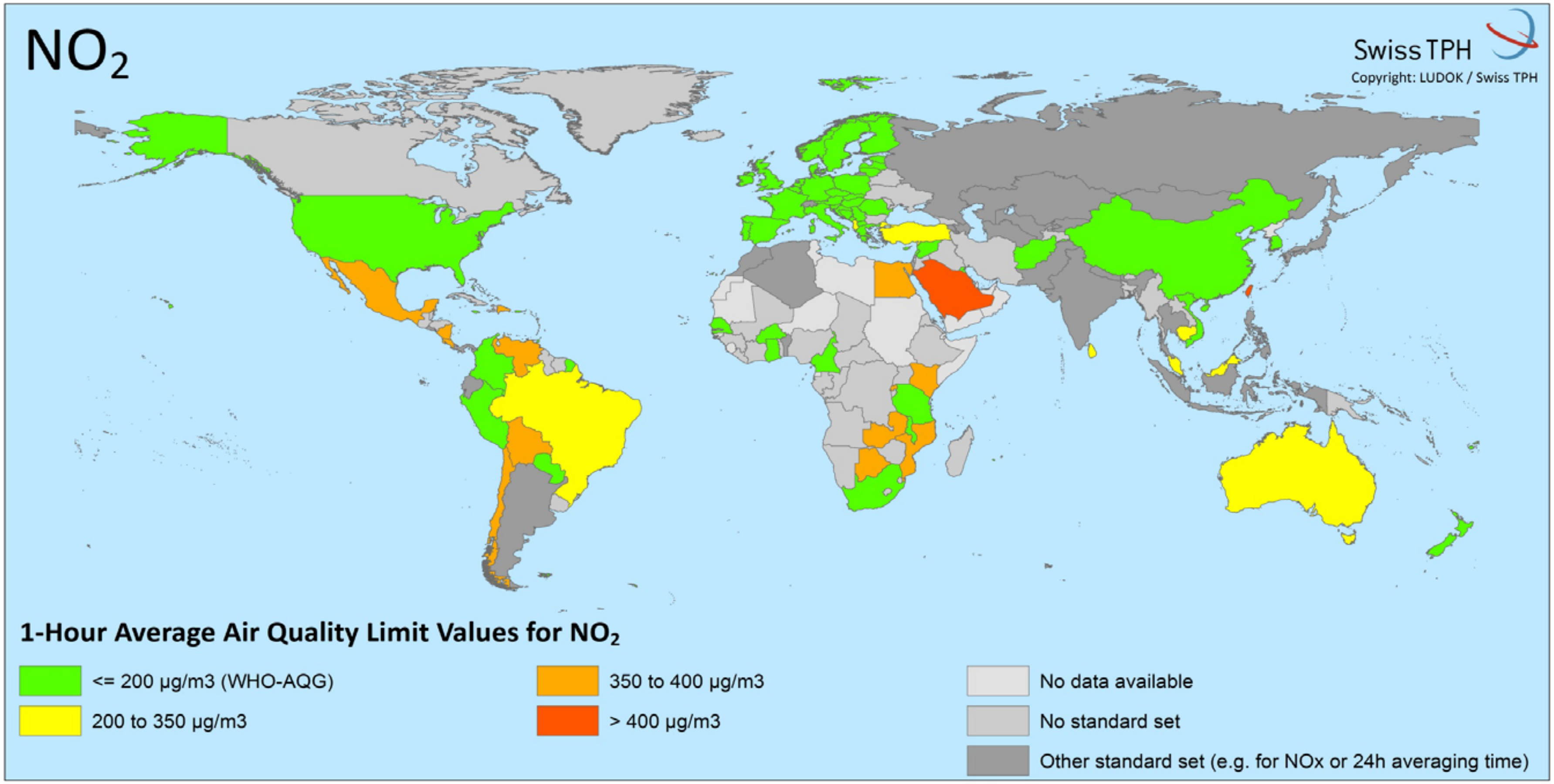

Online Resource 9. World map of national air quality standards for 8-hour maximum concentration of Ozone in relation to WHO air quality guideline values (WHO-AQG) and interim targets.

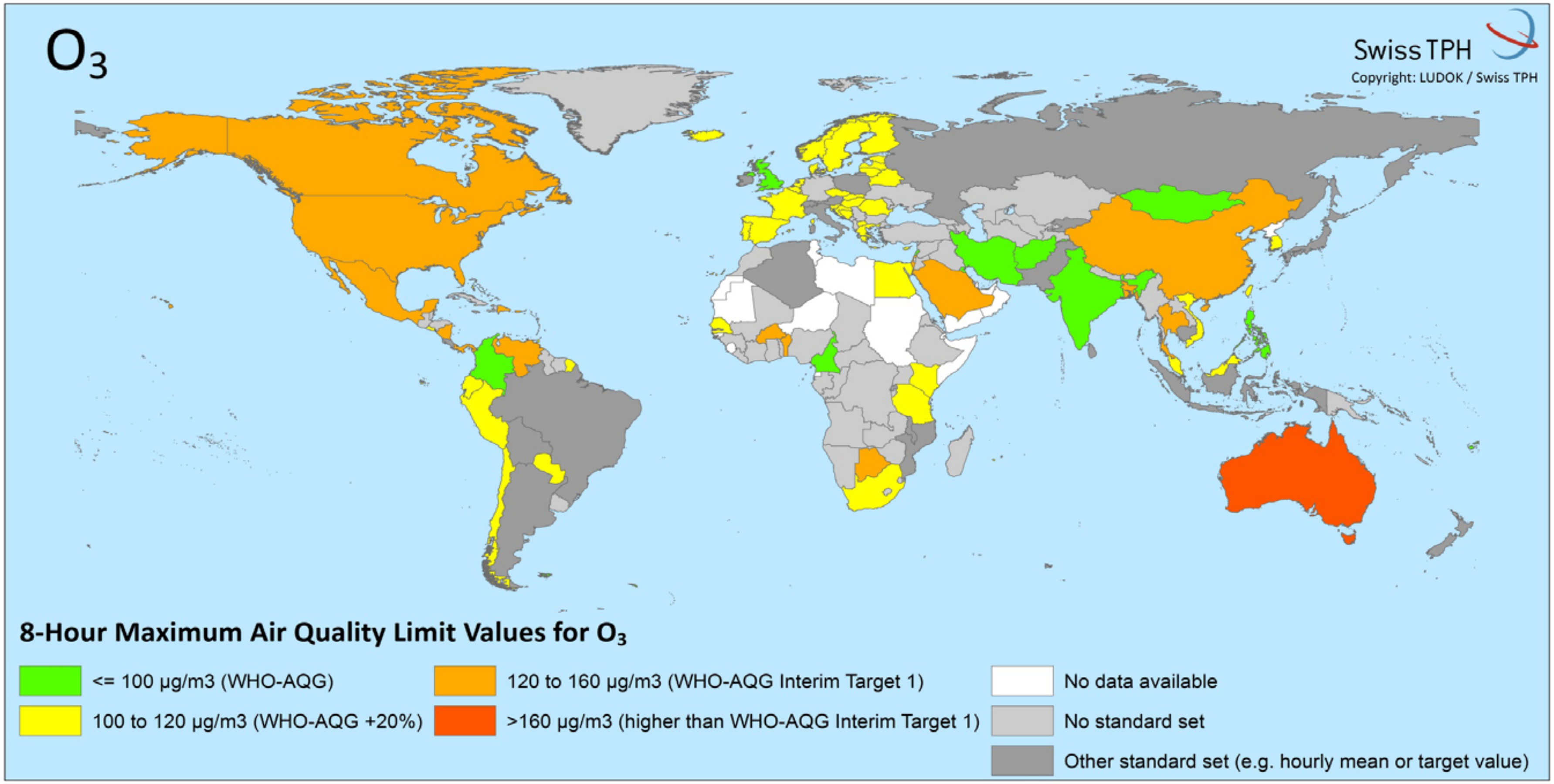

Online Resource 10. World map of national air quality standards for 8-hour average concentration of CO in relation to WHO air quality guideline values (WHO-AQG) and interim targets.

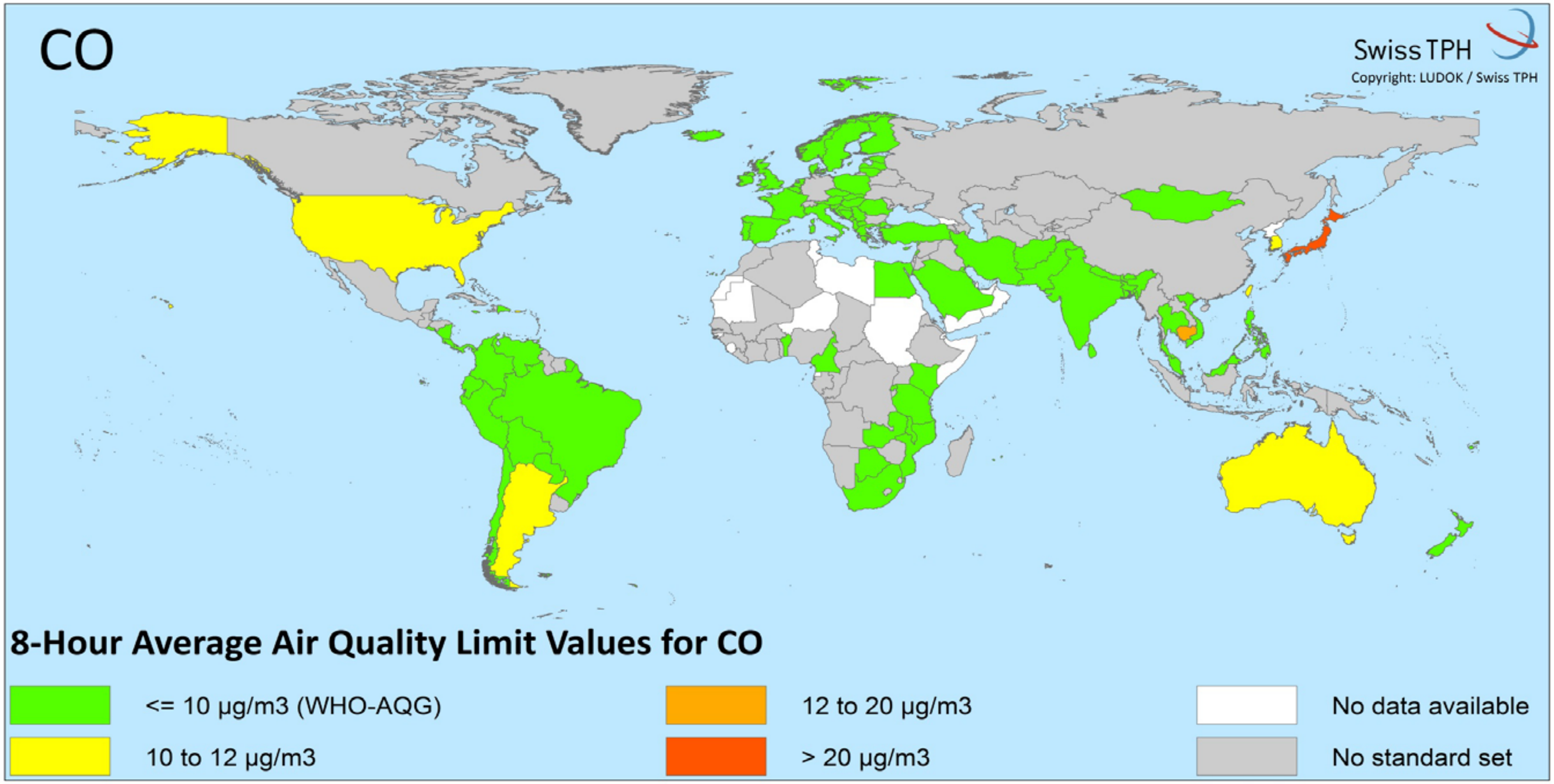

Online Resource 11. World map of national air quality standards for 1-hour average concentration of CO in relation to WHO air quality guideline values (WHO-AQG) and interim targets.

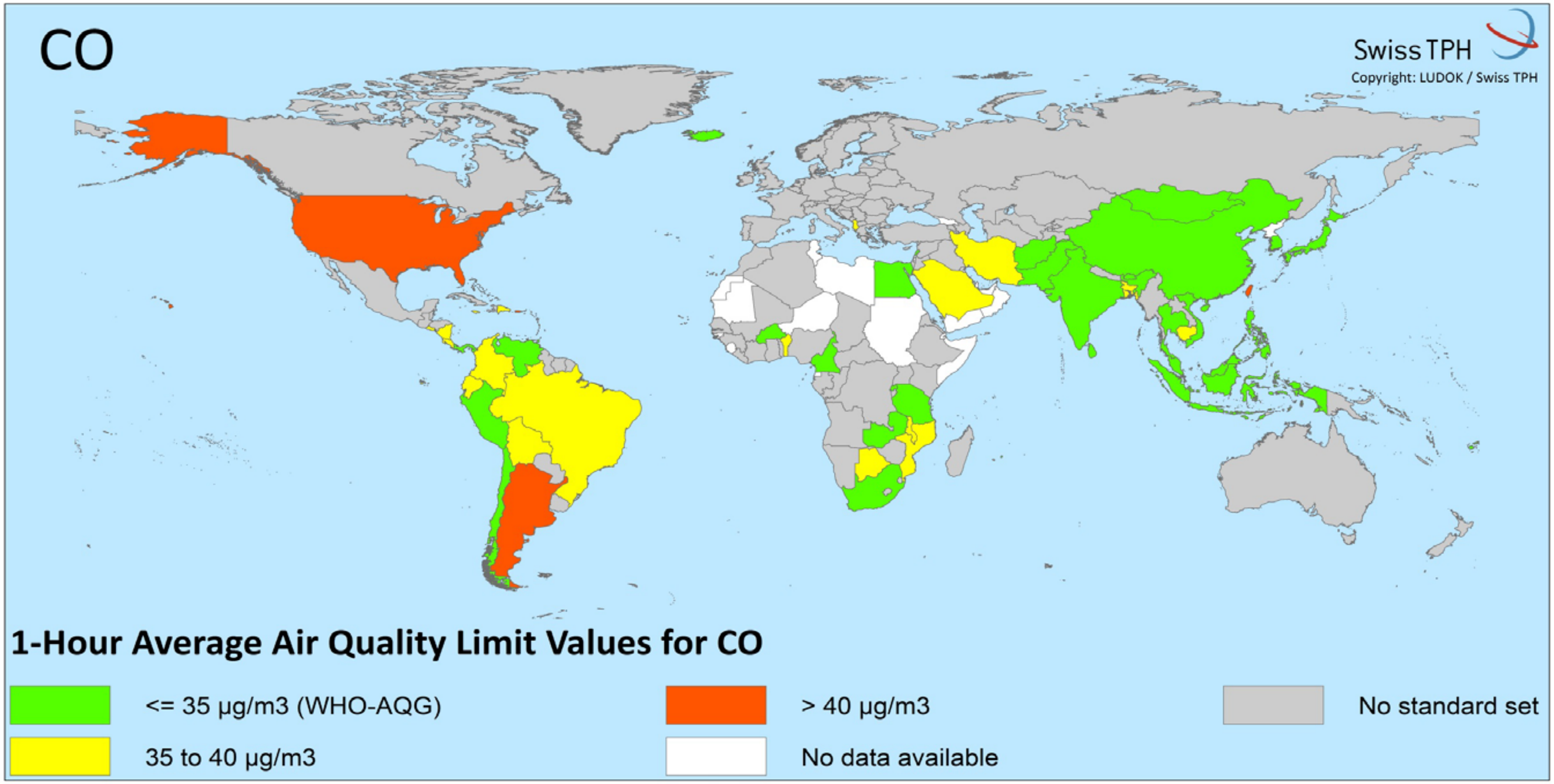

Online Resource 12. Existing long-term ambient air quality standards for Particulate Matter by WHO Regions in countries recognized by the United Nations and actual annual mean measurement data from the WHO Global Urban Ambient Air Pollution Database.

	PM10 standard annual mean	Measure ments: Highest	Measure ments: Lowest	Mean if >3 stations	Year of measurem ent	Difference highest vs standard	Measure ment sites per country
**WHO REGIONS AQSs**							
European Region							
**EU Directive 2008/50/EC**	40						
Austria	40	27	13	21	2013	-8	60
Belgium	40	31	13	24	2013	-11	43
Croatia	40	37	18	31	2013	-13	5
Cyprus	40	47	26	37	2013	-10	5
Czech Republic	40	45	18	28	2013	-10	49
Denmark	40	27	16	22	2013	-6	5
Estonia	40	17	14	16	2013	-2	4
Finland	40	19	4	14	2014	-9	24
France	40	36	7	19	2014	-12	315
Germany	40	28	8	19	2013	-11	161
Greece	40	42	22	34	2013	-12	10
Hungary	40	35	17	26	2013	-9	17
Ireland	40	22	12	16	2013	-5	8
Italy	40	47	6	26	2013	-20	236
Latvia	40	34	17	24	2013	-7	4
Lithuania	40	37	26	30	2013	-4	9
Luxemburg	40	22	16	20	2013	-4	3
Malta	40	38	23	28	2013	-6	4
The Netherlands	40	28	19	23	2013	-4	24
Poland	40	58	10	34	2013	-23	154
Portugal	40	21	9	15	2014	-6	12
Romania	40	44	12	25	2013	-13	42
Slovakia	40	40	22	29	2013	-8	21
Slovenia	40	30	20	26	2013	-6	13
Spain	40	33	6	18	2013	-12	225
Sweden	40	29	7	20	2013	-12	19
United Kingdom	40	31	12	18	2013	-6	51
Albania	50	32	32	32	2013	0	32
Andorra	40	18	18		2014	18	1
Bosnia and Herzegovina	40	106	50		2010	50	2
Iceland	20	15	8	12	2013	-3	3
Israel	50	57	31	44	2014	-13	40
Montenegro	40	77	26	42	2014	-16	5
Norway	25	28	16	20	2013	-5	12
Russian Federation	40	33	33		2009	33	1
Serbia	40	37	31	34	2013	-3	3
Switzerland	20	23	16	20	2013	-4	9
oslav Republic of Macedonia	40	140	51	83	2013	-32	4

Turkey	60	109	12	61	2012	-49	81
Region of the Americas						0	
olivia (Plurinational State of)	50	82	60		2014	60	2
Brazil	50	95	12	34	2014	-22	45
Chile	50	75	12	46	2014	-35	23
Colombia	50	52	18	40	2014	-22	18
Costa Rica	50	47	20	29	2013	-9	7
Ecuador	50	69	18	36	2012	-17	9
El Salvador	50	77	77		2014	77	1
Jamaica	50	54	24	38	2012	-14	6
Mexico	40	86	32	56	2011	-24	9
Panama	50	31	31		2013	31	1
Peru	50	88	88		2013	88	1
:uela (Bolivarian Republic of)	50	47	47		2012	47	1
African Region						0	
Cameroon	20	141	65	104	2012	-39	3
Senegal	80	141	141		2013	141	1
South Africa	50	119	24	54	2014	-30	13
United Republic of Tanzania	60	35	35		2011	35	1
Afghanistan	20	334	260		2009	260	2
Iran (Islamic Republic of)	20	527	18	115	2010	-97	25
Jordan	70	128	45	77	2015	-32	4
Kuwait	90	212	63	139	2014	-76	11
Pakistan	120	540	198	339	2011	-141	5
Saudi Arabia	80	368	153	248	2014	-95	7
Bangladesh	50	191	64	140	2014	-77	8
Bhutan	60	78	78		2012	78	1
India	60	329	11	107	2012	-96	122
Sri Lanka	50	64	64		2011	64	1
Thailand	50	57	23	45	2014	-22	26
**Western pacific**						0	
China	50	305	16	84	2014	-69	210
Malaysia	50	47	20	31	2014	-11	6
Mongolia	50	165	165		2010	165	1
Philippines	60	84	21	52	2013	-31	8
Republic of Korea	50	54	38	47	2014	-9	16

Reference: WHO (2016a) WHO Global Urban Ambient Air Pollution Database (update 2016). WHO: Public health, environmental and social determinants of health (PHE). http://www.who.int/phe/health_topics/outdoorair/databases/cities/en/. Accessed 13.7.2016 2016

Online Resource 13. Comparison of national annual air quality standard set for PM10 to measured annual mean concentrations for the year 2013.

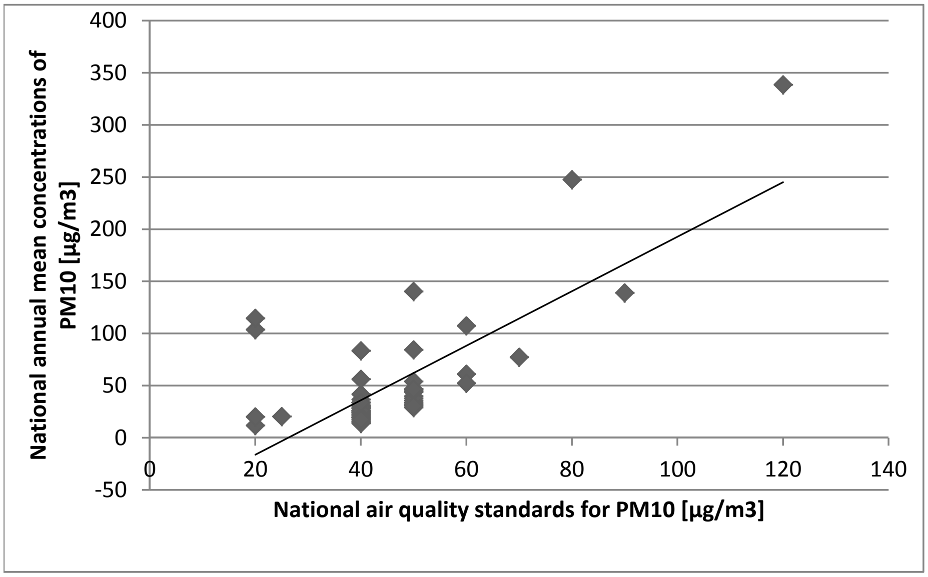

Online Resource 14. Comparison of national annual air quality standard set for PM10 to highest measurement of annual concentration of PM10 in 2013.

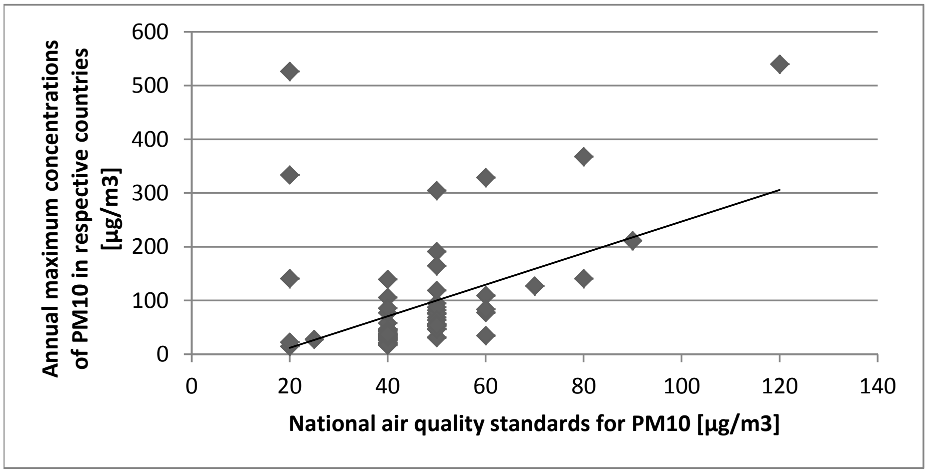

Supplement: Supplementary file 1 — Supplementary material 1 (PDF 5776 KB) [file 38_2017_952_MOESM1_ESM.pdf]
